# A Competition between Stimulators and Antagonists of Upf Complex Recruitment Governs Human Nonsense-Mediated mRNA Decay

**DOI:** 10.1371/journal.pbio.0060111

**Published:** 2008-04-29

**Authors:** Guramrit Singh, Indrani Rebbapragada, Jens Lykke-Andersen

**Affiliations:** Molecular, Cellular, and Developmental Biology, University of Colorado at Boulder, Boulder, Colorado, United States of America; University of Wisconsin, United States of America

## Abstract

The nonsense-mediated decay (NMD) pathway subjects mRNAs with premature termination codons (PTCs) to rapid decay. The conserved Upf1–3 complex interacts with the eukaryotic translation release factors, eRF3 and eRF1, and triggers NMD when translation termination takes place at a PTC. Contrasting models postulate central roles in PTC-recognition for the exon junction complex in mammals versus the cytoplasmic poly(A)-binding protein (PABP) in other eukaryotes. Here we present evidence for a unified model for NMD, in which PTC recognition in human cells is mediated by a competition between 3′ UTR–associated factors that stimulate or antagonize recruitment of the Upf complex to the terminating ribosome. We identify cytoplasmic PABP as a human NMD antagonizing factor, which inhibits the interaction between eRF3 and Upf1 in vitro and prevents NMD in cells when positioned in proximity to the termination codon. Surprisingly, only when an extended 3′ UTR places cytoplasmic PABP distally to the termination codon does a downstream exon junction complex enhance NMD, likely through increasing the affinity of Upf proteins for the 3′ UTR. Interestingly, while an artificial 3′ UTR of >420 nucleotides triggers NMD, a large subset of human mRNAs contain longer 3′ UTRs but evade NMD. We speculate that these have evolved to concentrate NMD-inhibiting factors, such as PABP, in spatial proximity of the termination codon.

## Author Summary

The nonsense-mediated mRNA decay pathway is responsible for rapidly degrading mRNAs with premature termination codons. This is important because it prevents the production of potentially deleterious truncated proteins from aberrant mRNAs, such as those that have undergone erroneous processing. How does the cell discriminate aberrant mRNAs from those that are normal? Here we present evidence that in human cells, the targeting of an mRNA to nonsense-mediated mRNA decay depends on a competition between proteins associated with the mRNA 3′ UTR that stimulate or antagonize mRNA decay. We show that cytoplasmic poly(A)-binding protein, a protein associated with the mRNA 3′ end poly(A) tail, antagonizes mRNA decay. By contrast, a protein complex deposited onto mRNAs upon pre-mRNA splicing, called the exon junction complex, stimulates mRNA decay. Our observations suggest that the competition between these proteins, and probably other unknown proteins with similar activities, determines whether a key protein complex in the pathway, the Upf complex, is recruited to the mRNA upon translation termination, which leads to mRNA decay.

## Abstract 3

Human mRNAs with premature termination codons are detected and degraded by nonsense-mediated decay when 3' untranslated region-associated proteins, such as poly(A)-binding protein, are absent from the proximity of the terminating ribosome.

## Introduction

The process of nonsense-mediated decay (NMD) subjects mRNAs with premature termination codons (PTCs) to rapid decay. This helps rid the cell of aberrant mRNAs that have acquired PTCs through mutation or faulty processing [1–3]. Moreover, several lines of evidence suggest that NMD is also used as a posttranscriptional mechanism of normal gene regulation [4]. The NMD pathway employs a set of factors that are conserved amongst eukaryotes. Central to the NMD pathway is the Upf complex, which consists of the proteins Upf1, Upf2, and Upf3 [1–3]. The Upf complex interacts with the eukaryotic translation release factors, eRF3 and eRF1, and triggers NMD when translation termination takes place at a PTC [1–3]. In addition, the Smg proteins, which are conserved in metazoans, regulate Upf1 function by phosphorylation and dephosphorylation [2,3].

A fundamental question is how mRNAs with PTCs are distinguished from those with normal termination codons. Despite the conservation of core NMD factors, contrasting models have been proposed in mammalian cells as opposed to other eukaryotes. Evidence in Saccharomyces cerevisiae and in cell lines from Drosophila melanogaster suggests that termination codons are recognized as PTCs when positioned too far upstream of the poly(A) tail [5–7]. This is thought to be a consequence of an impaired interaction between eRF3 at the terminating ribosome and factors associated with the normal 3′ UTR, including cytoplasmic poly(A)-binding protein (PABP) [1,5,7], which on mRNAs with regular stop codons (proximal to the poly(A) tail) stimulates normal translation termination [8]. Consistent with this model for NMD, termed the “faux 3′ UTR” model [1,7], 3′ UTRs of S. cerevisiae and D. melanogaster mRNAs are generally short, on average ∼100 and ∼330 nucleotides in length, respectively [9,10]. Interestingly, recent observations show evidence that cytoplasmic PABP is not required for the discrimination of normal termination codons from PTCs in S. cerevisiae [11]. Thus, cytoplasmic PABP may function redundantly with other 3′ UTR–associated factors to antagonize NMD.

3′ UTRs of human mRNAs are on average longer (∼750–800 nucleotides [12]) than those of S. cerevisiae and D. melanogaster, and current models for NMD in mammalian cells do not involve the length of the 3′ UTR. Rather, the exon junction complex (EJC), which is deposited 20–25 nucleotides upstream of mRNA exon-exon junctions after pre-mRNA splicing [13], is thought to play a central role. A termination event more than ∼30 nucleotides upstream of one or more EJCs is thought to trigger NMD through EJC-mediated recruitment of the Upf complex [2,3]. This is consistent with observed direct interactions between EJC components and Upf3 proteins [14–18]. However, the EJC plays no apparent role in NMD in D. melanogaster [19] or in Caenorhabditis elegans [20] and no evidence for the existence of an EJC has been reported in yeast. Nevertheless, a conceptually similar model to the EJC model was proposed earlier for NMD of the PGK1 mRNA in yeast, in which a “downstream sequence element” (DSE), when present downstream of a termination codon, promotes NMD through recruitment of the protein Hrp1p, which interacts with Upf proteins [21,22].

A fundamental difference between the faux 3′ UTR and the EJC/DSE models for NMD is that the EJC/DSE models propose that NMD-stimulating factors (the EJC and Hrp1p, respectively) trigger NMD when positioned downstream of a termination codon, whereas the faux 3′ UTR model postulates that NMD is caused instead by the absence of NMD-antagonizing factors, such as cytoplasmic PABP, which normally positively influence translation termination and mRNA stability. Here, we present evidence for a merged model for NMD in human cells, which likely can be extended to other eukaryotes. According to this model, PTC recognition is determined by a competition between 3′ UTR–associated factors, which stimulate (including the EJC) or antagonize (including cytoplasmic PABP) the recruitment of the Upf complex to the terminating ribosome. Our observations suggest that the fundamental principles of the NMD pathway are much more conserved between mammals and other eukaryotes than previously anticipated.

## Results

### 3′ UTR Introns Are Not Sufficient for Triggering Human NMD

**Figure 1:**
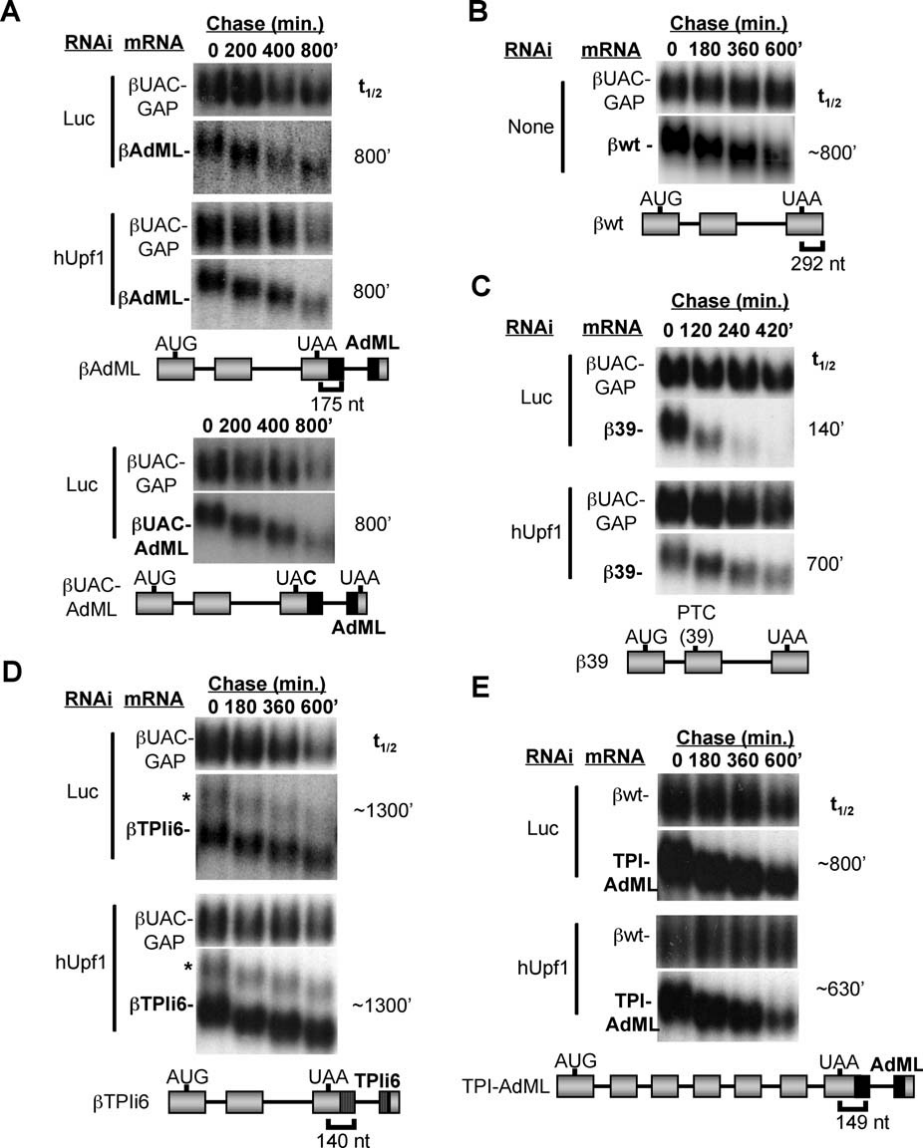
A 3′ UTR Intron Is Not Sufficient to Trigger NMD (A–E) Decay assays in human HeLa Tet-off cells for β-globin (A,D) or TPI mRNAs (E) with inserted 3′ UTR introns or β-globin wild-type (B) or PTC-39 mutant (C) mRNAs in the presence of Luciferase (Luc) or hUpf1 siRNAs as indicated (knockdown efficiencies are shown in Figure S1). For each panel, schematics are shown for each tested mRNA below the panels; β-globin or TPI exons are indicated as light-gray bars, introns as lines, AdML exons are shown in black, and TPIi6 exons are patterned. Numbers above the panels indicate time after transcriptional repression. mRNA half-lives were calculated by comparison with the constitutively expressed internal control mRNAs (βUAC-GAP or βwt; top panels in each assay) and are given on the right.

The EJC model for human NMD postulates that any translation termination event taking place >50–55 nucleotides upstream of an exon-exon junction should result in NMD. However, during our studies of the human NMD pathway, we observed that a β-globin mRNA, in which the adenovirus major late (AdML) intron was inserted into the 3′ UTR 175 nucleotides downstream of the normal β-globin mRNA translation termination codon, did not show enhanced mRNA decay as compared to the wild-type β-globin mRNA in human HeLa Tet-off cells (compare Figure 1A and 1B). Moreover, in contrast to a well-characterized β-globin NMD substrate, which contains a premature termination codon at position 39 (β39; Figure 1C), the AdML intron containing β-globin mRNA is not stabilized when the central NMD factor hUpf1 is knocked down (Figure 1A, middle panel and Figure S1) or when a point mutation causes translation termination to take place downstream of the inserted intron (Figure 1A, bottom panel). Sequencing of cDNAs derived from the mRNAs in Figure 1A revealed the expected splicing patterns (unpublished data). Thus, in contrast to the prediction from the EJC model for human NMD, the AdML intron is not sufficient for triggering NMD when positioned in the 3′ UTR of β-globin mRNA, even though the AdML intron has been previously demonstrated to recruit an EJC [13].

The observation in Figure 1A was surprising, because a β-globin mRNA in which the MINX-intron had been placed in the 3′ UTR was previously found to cause reduced mRNA steady-state levels [23]. Therefore, to rule out the possibility that the observations in Figure 1A represent an unusual property of the specific mRNA reporter, we tested two other substrates. As seen in Figure 1D and 1E, insertion of the triosephosphate isomerase (TPI) mRNA intron 6 or the AdML intron, 140 or 149 nucleotides downstream of the termination codons of β-globin or TPI mRNAs, respectively, failed to cause hUpf1-dependent mRNA decay, despite the previously demonstrated ability of each of these introns to recruit an EJC [13]. Sequencing of cDNAs derived from the tested mRNAs revealed the expected splicing patterns (unpublished data), although a minor fraction of the βTPIi6 mRNA fails to remove the TPI intron (see asterisk in Figure 1D). We attempted to test the β-globin mRNA with the MINX-intron in the 3′ UTR, which was previously found to accumulate at reduced steady-state levels as compared to wild-type β-globin mRNA [23] (construct generously provided by A. Kulozik and M. Hentze). However, the MINX intron (as well as a number of other introns tested in this study) failed to be spliced out of the β-globin mRNA 3′ UTR in the HeLa Tet-off cells used here (unpublished data). We conclude that a 3′ UTR intron is not sufficient to trigger NMD in human cells. This contradicts an EJC-centric model for human NMD.

### Extended 3′ UTRs Trigger Human NMD

**Figure 2:**
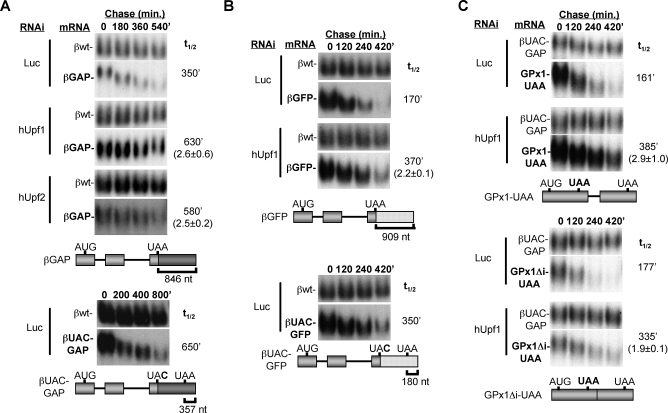
Intron-Less Extended 3′ UTRs Trigger NMD in Human Cells (A and B) mRNA decay assays showing decay rates of β-globin-derived βGAP, βUAC-GAP, βGFP, and βUAC-GFP mRNAs (see schematics below) in human HeLa Tet-off cells expressing siRNAs against hUpf1, hUpf2, or as a negative control, Luciferase (Luc), as indicated on the left of each panel (knockdown efficiencies are shown in Figure S1). Constitutively expressed βwt mRNA was used as internal controls for quantification. Numbers above the panels indicate time after transcriptional repression. Schematics on the bottom show the used constructs with β-globin exons indicated as light-gray bars, introns as lines, and GAPDH and GFP sequences as dark-gray and dotted bars, respectively. Numbers on the right indicate mRNA half-lives (t_1/2_; in minutes) calculated from the shown experiment. Numbers in parentheses indicate the fold stabilization with standard deviation (*n* ≥ 3) upon hUpf knockdown as compared to the Luc control. (C) mRNA decay assays for intron-containing GPx1 mRNA with a PTC (GPx1-UAA) or a GPx1 mRNA with a PTC expressed from an intron-less construct (GPx1Δi-UAA) (see schematics below) in the presence of Luc or hUpf1 siRNAs as indicated on the left. mRNA decay rates for the shown experiments are given on the right and numbers in parentheses indicate the fold stabilization with standard deviation (*n* ≥ 3) upon hUpf knockdown as compared to the Luc control.

Our observation that 3′ UTR introns are not sufficient for triggering human NMD spurred us to test whether cytoplasmic PABP may antagonize NMD in human cells, as it does in S. cerevisiae and D. melanogaster. We therefore first manipulated the position of the poly(A) tail relative to the termination codons of β-globin and TPI reporter mRNAs and tested the effect on mRNA decay. As seen in Figure 2A and 2B and Figure S2, artificial extension of the 3′ UTRs of β-globin or TPI mRNAs (from 292 and 447 nucleotides, respectively, to 846–1,112 nucleotides) through insertion of fragments of glyceraldehyde 3-phosphate dehydrogenase (GAPDH) (Figures 2A and S2) or green fluorescent protein (GFP) (Figure 2B) mRNAs results in mRNA destabilization (compare with Figures 1B and S2, top panel). This is due to NMD because depletion of the NMD factors hUpf1 or hUpf2 stabilizes the mRNAs (Figure 2A and 2B and Figures S1 and S2). Moreover, the introduction of single point mutations in the termination codons that results in termination on the same mRNAs in proximity (180–357 nucleotides upstream) of the poly(A) tail, results in mRNA stabilization (Figure 2A and 2B and S2, bottom panels). Even though sequencing of cDNAs derived from the tested mRNAs revealed no cryptic splicing in the extended 3′ UTRs (unpublished data), depletion of the central EJC component eIF4AIII results in stabilization of the βGAP mRNA (Figure S3), possibly reflecting the ability of the EJCs in the β-globin mRNA open reading frame to stimulate translation as has been previously observed [24,25]. Successive shortening of the 3′ UTR of the βGAP mRNA revealed that a 3′ UTR as short as 422 nucleotides can trigger NMD (Figure S4). This is surprising because a large fraction of human mRNAs contain 3′ UTRs longer than 422 nucleotides [12]. We conclude that artificially extended 3′ UTRs trigger NMD in human cells. This is consistent with recent reports in which steady-state levels of PTCs containing TPI, β-globin, and Ig-μ reporter mRNAs lacking 3′ UTR introns were measured [26–28], and with observations using unspliced Rous sarcoma virus RNAs in chicken cells [29]. Thus 3′ UTR introns are neither necessary (Figure 2) nor sufficient (Figure 1) for human NMD.

### An Intron-Less mRNA Can Undergo NMD

Having observed that 3′ UTR introns are not required for NMD, we asked whether a completely intron-less mRNA can undergo NMD. It was observed previously that introduction of PTCs in the naturally intron-less Hsp70 and histone H2A mRNAs does not result in their decreased steady-state levels, which led to speculations that intron-less mRNAs are immune to NMD [30]. However, it has been pointed out that wild-type Hsp70 and histone H2A mRNAs are both highly unstable and may thus not be further destabilized by a PTC [5]. We therefore tested the stability of three naturally occurring intron-less mRNAs (encoding eRF3b, SFN, and TBCC) and found that both wild-type and PTC containing versions of these mRNAs were unstable (∼100- to 150-min half-lives, unpublished data). Thus, mRNA instability may be a general feature of natural intron-less mRNAs. However, when the only intron in the Glutathione Peroxidase 1 (GPx1) mRNA is removed, introduction of a PTC triggers NMD, although not as efficiently as in the presence of the intron (Figure 2C, compare lower and upper panels). Thus, neither 3′ UTR introns nor internal introns are essential for human NMD. However, similarly to a previous report [30] we have not been able to identify a natural human intron-less mRNA for which NMD could be observed, perhaps due to the observed inherent instabilities of the tested mRNAs.

### Cytoplasmic PABP Antagonizes Human NMD

**Figure 3:**
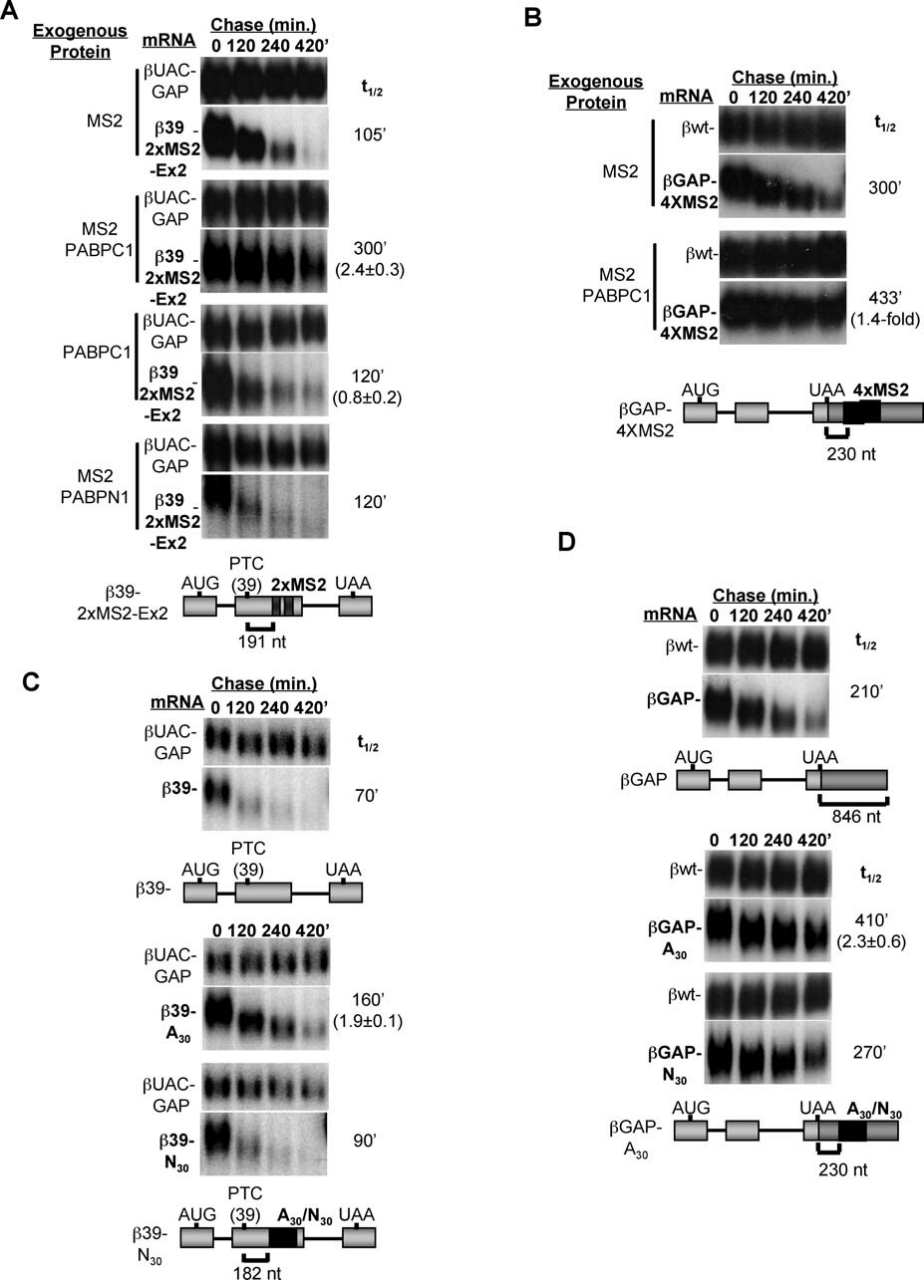
PABPC1 Antagonizes NMD (A and B) mRNA decay assays for β39–2xMS2-Ex2 and βGAP-4xMS2 mRNAs (see schematics below; MS2 binding sites indicated as black bars and GAPDH sequence in dark gray) in HeLa Tet-off cells expressing the MS2 coat protein fused to PABPC1 (MS2-PABPC1) or PABPN1 (MS2-PABPN1) or individual unfused proteins as indicated on the left of the panels. Constitutively expressed βUAC-GAP or βwt mRNAs were used as internal controls (upper panels). (C and D) Northern blots showing decay rates of β39 and βGAP mRNAs, with or without A_30_ or N_30_ sequences inserted downstream of the PTCs (schematic below). For all panels (A–D), numbers on the right indicate mRNA half-lives (t_1/2_; in minutes) calculated from the shown experiments, and numbers in parentheses indicate the fold stabilization with standard deviation (*n* ≥ 3) as compared with the control experiments shown in the corresponding top panels.

To more directly test whether cytoplasmic PABP antagonizes NMD in human cells, we examined the effect of positioning cytoplasmic PABP in proximity of a PTC using two different approaches. First, as seen in Figure 3A and 3B, when an MS2-PABPC1 fusion protein (PABPC1 is one of five human cytoplasmic PABPs [31]) is artificially tethered downstream of a PTC in two different β-globin NMD reporter mRNAs, a partial rescue of NMD is observed. This rescue is due to tethered PABPC1, because similar levels of unfused PABPC1 (Figure 3A) or MS2 coat protein (Figure 3A and 3B) do not stabilize the mRNAs. Moreover, tethering of the nuclear poly(A)-binding protein PABPN1 does not rescue NMD (Figure 3A) even though it is expressed at levels similar to MS2-PABPC1 (Figure S5). The efficiency of the rescue from NMD by tethered PABPC1 decreases as the MS2 binding sites are moved more distal to the PTC in the β-globin PTC-39 mRNA (Figure S6).

As a second independent approach to ask whether PABP can antagonize human NMD, we tested the effect of inserting a binding site for PABP downstream of the PTCs. As seen in Figure 3C and 3D, inclusion of a poly-A_30_-stretch, but not that of a random 30-nucleotide stretch, 182 or 230 nucleotides downstream of the PTC of two different NMD reporter mRNAs, results in partial rescue of NMD. Thus, similarly to S. cerevisiae [7] and D. melanogaster [5], cytoplasmic PABP can antagonize NMD in human cells when placed in proximity of a PTC. Recent observations suggest that while cytoplasmic PABP can antagonize NMD in S. cerevisiae, it is not required for discriminating a normal mRNA from an NMD substrate [11]. Attempts at testing whether PABPC1 is required for preventing NMD in human cells failed because HeLa Tet-off cells became detached from plates upon short interfering RNA (siRNA)-mediated PABPC1 depletion (unpublished data).

### A Subset of Naturally Occurring Long 3′ UTRs Can Antagonize NMD

**Figure 4:**
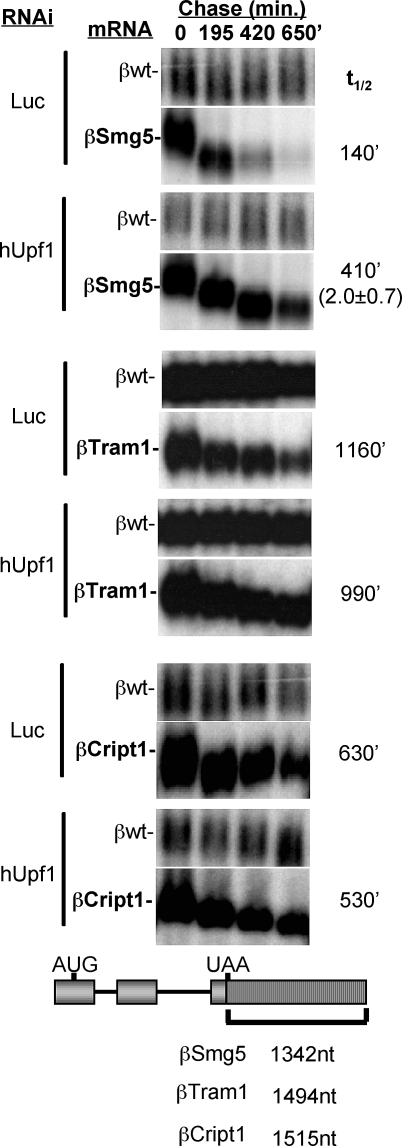
Normal Long 3′ UTRs Can Evade NMD mRNA decay assays for βSmg5, βTram1, and βCript1 mRNAs (schematics with the respective 3′ UTR lengths are shown below) in the presence of Luc or hUpf1 siRNAs. mRNA half-lives are given on the right. The number in parentheses for the βSmg5 mRNAs indicates the fold stabilization with standard deviation (*n* ≥ 3) upon hUpf knockdown as compared to the Luc control.

Our observations raise the question of whether naturally occurring mammalian mRNAs with long 3′ UTRs, which can be several kilobases in length, are normal targets of NMD or whether they have evolved mechanisms to evade the NMD pathway. We noted that mRNAs identified by microarray assays to be upregulated upon hUpf1 knockdown in HeLa cells [32] contain on average significantly longer 3′ UTRs than those mRNAs unaffected by hUpf1 knockdown (Figure S7). Moreover, the majority of these 3′ UTRs (75%) are longer than the ∼420 nucleotides observed here to trigger NMD in the βGAP reporter mRNA (Figures S4 and S7). It is possible that at least a subset of these transcripts undergo NMD due to an increased distance between the termination codon and the poly(A) tail. Indeed, when the 1,342-nucleotide 3′ UTR of one of these mRNAs, encoding hSmg5, is replaced for the β-globin 3′ UTR (βSmg5), the chimeric mRNA undergoes NMD (Figure 4, top two panels). Thus, the Smg5 3′ UTR stimulates NMD, and a subset of mRNAs may have evolved long 3′ UTRs to be regulated by the NMD pathway. However, numerous mRNAs with long 3′ UTRs are not upregulated upon hUpf1 knockdown [32] (Figure S7). When the 3′ UTRs from two such mRNAs, Cript1 and Tram1, were inserted into the β-globin mRNA, no NMD was observed (Figure 4, bottom panels). This is in sharp contrast to the observations using artificial long 3′ UTRs (compare to Figures 1 and S4) and suggests that the ability of a subset of endogenous long 3′ UTRs to evade NMD is an acquired property (see Discussion).

### A 3′ UTR Intron Can Enhance Human NMD

**Figure 5:**
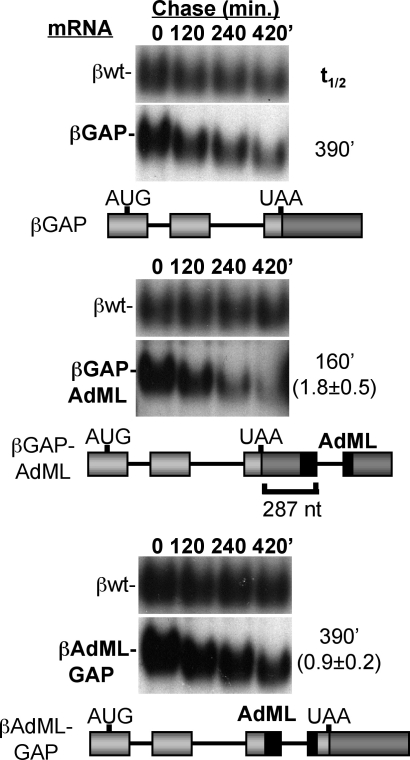
A 3′ UTR Intron Enhances NMD Decay assays for βGAP mRNA in the absence or presence of the AdML intron inserted into the GAPDH sequence or exon 3 as indicated. For each panel, schematics are shown for each tested mRNA below the panels; AdML exons are shown in black. mRNA half-lives are given on the right with the fold enhancement of mRNA decay rates and standard deviation (*n* ≥ 3) as compared with the control experiment in the top panel shown in parentheses.

Our observations that 3′ UTR introns are neither necessary (Figure 2) nor sufficient (Figure 1) for human NMD raises the question of whether introns play any role in human NMD. We therefore tested the effect of inserting the AdML intron into the 3′ UTR of an mRNA, which already undergoes NMD due to an extended 3′ UTR. Interestingly, insertion of the AdML intron downstream of the termination codon of βGAP mRNA results in enhanced mRNA decay (Figure 5; βGAP-AdML mRNA). This effect is only observed when the intron is positioned in the 3′ UTR, as insertion of the same intron upstream of the termination codon (without disrupting the open reading frame) does not enhance mRNA decay (Figure 5; βAdML-GAP mRNA). Thus, while a downstream intron is neither sufficient nor necessary for triggering NMD in human HeLa cells (Figures 1 and 2) [33], it can enhance the degradation of an mRNA that is already a target of NMD due to an extended 3′ UTR (Figure 5A and 5B). Consistent with this, the presence of an intron appears to also stimulate NMD of a PTC containing GPx1 mRNA (Figure 2C).

### PABPC1 Can Out-Compete the Interaction between hUpf1 and eRF3 In Vitro

**Figure 6:**
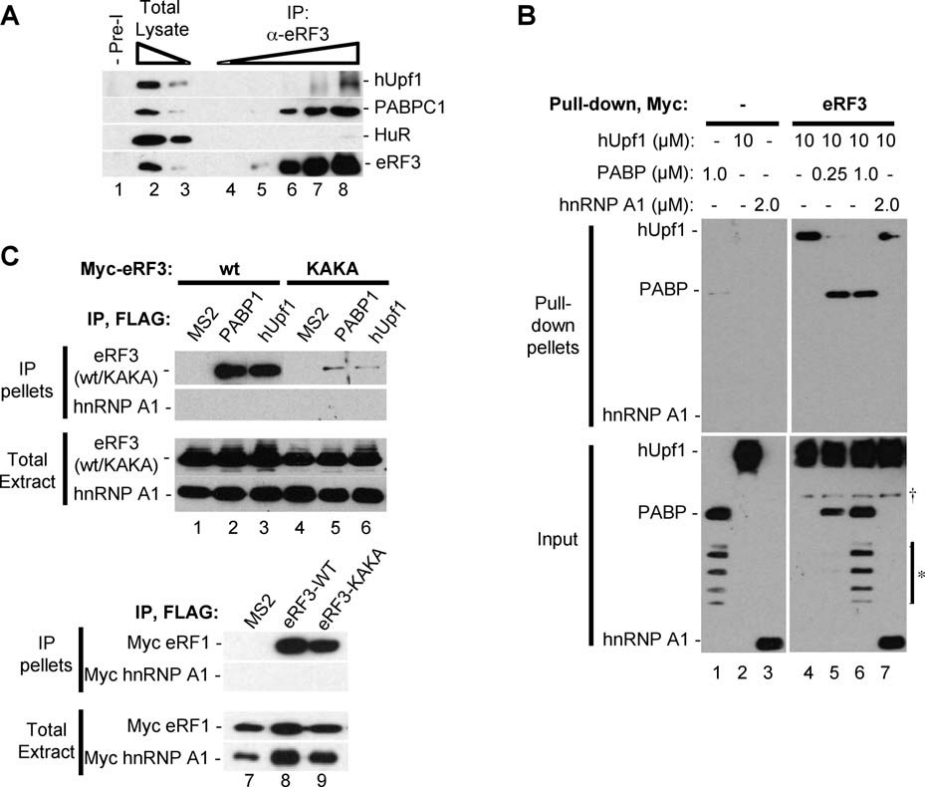
PABPC1 Antagonizes the Interaction between eRF3 and hUpf1 In Vitro (A) Co-IP assays showing the co-IP of endogenous hUpf1 and PABPC1 and HuR proteins with an antibody against eRF3 (α-eRF3; 1%, 3%, 10%, 30%, and 100% of pellet loaded in lanes 4–8, respectively), or pre-immune serum (Pre-I, lane 1) as a control. 3% and 0.6% of the total lysate are shown in lanes 2 and 3, respectively. (B) In vitro pull-down assays showing anti-FLAG Western blots of pull-down pellets (upper panels) resulting after myc-tagged eRF3 (lanes 4–7) or no myc-tagged protein (lanes 1–3), immobilized on an anti-myc antibody resin, was incubated with various amounts of FLAG-tagged hUpf1, PABPC1, or hnRNP A1 as indicated. Estimated amounts of FLAG-tagged proteins in each reaction are given in μM. Bottom panels show 5% of input protein for each reaction. The asterisk (*) and dagger (†) on the right indicates likely degradation products of PABPC1 and cross-reacting Myc-eRF3, respectively. (C) Lanes 1–6: Western blot for exogenously expressed Myc-tagged eRF3 (wt, lanes 1–3) or eRF3 KAKA mutant protein (KAKA, lanes 4–6) that co-IP with FLAG-tagged PABPC1, hUpf1, or as a negative control, MS2, as indicated above the lanes. Lanes 7–9: Western blot for exogenously expressed Myc-tagged eRF1 that co-IP with FLAG-tagged eRF3, eRF3 KAKA mutant protein, or as a negative control, MS2, as indicated above the lanes. For all lanes, 5% of total input extracts are shown in the bottom panels.

How does cytoplasmic PABP antagonize NMD when positioned in proximity of the termination codon? Both cytoplasmic PABP and Upf1 have been previously observed to stimulate translation termination in yeast cells [8,34] and to associate with translation release factor eRF3 [35–40]. This raised the possibility that cytoplasmic PABP inhibits NMD by preventing Upf1 from interacting with eRF3 and the terminating ribosome. As seen in the co-immunoprecipitation (co-IP) assays in Figure 6A, endogenous hUpf1 and PABPC1 can both be observed in complex with eRF3 in RNase-treated HeLa cell extracts. However, PABPC1 co-IPs much more efficiently than hUpf1 with eRF3 (Figure 6A), in spite of comparable estimated number of molecules of cytoplasmic PABP (∼8 × 10^6^/cell) and hUpf1 (∼3 × 10^6^/cell) in HeLa cells [41,42]. Consistent with this, bacterially expressed GST-tagged eRF3 was found to associate much more efficiently with rabbit reticulocyte-lysate-translated PABPC1 (K_d_ ∼ 5 nM) than hUpf1 (K_d_ > 1 μM) (unpublished data).

To test whether PABPC1 can antagonize the interaction between eRF3 and hUpf1 in vitro, we immunopurified transiently expressed epitope-tagged eRF3, PABPC1, and hUpf1 proteins from HEK 293T cells and tested the ability of hUpf1 to associate with eRF3 in the presence of increasing amounts of PABPC1. As seen in Figure 6B, in contrast to the negative control protein hnRNP A1, increasing amounts of PABPC1 efficiently prevent the interaction between hUpf1 and eRF3, even when hUpf1 is present in 10- to 40-fold excess over PABPC1 (Figure 6B, compare lanes 5 and 6 with lanes 4 and 7). Thus, PABPC1 can antagonize the interaction between hUpf1 and eRF3 in vitro. However, no reduction in the co-IP efficiency between hUpf1 and eRF3 was observed upon transient over-expression of FLAG-tagged PABPC1 in HeLa or HEK 293T cells (unpublished data). Thus, either exogenous PABPC1 failed to express at adequate levels to observe a competition in cells, or the relation between hUpf1, PABPC1, and eRF3 is more complex in cells than it is in vitro.

To test whether amino acid residues of eRF3, which are important for cytoplasmic PABP interaction, are also important for the interaction with hUpf1, we constructed a eRF3 protein (eRF3 KAKA) mutated in four N-terminal residues that are conserved between cytoplasmic PABP-binding proteins [38,43]. As seen in the co-IP assays in Figure 6C, the exogenously expressed eRF3 KAKA mutant protein is equally impaired in interaction with PABPC1 and hUpf1 (Figure 6C, compare lanes 5 and 6 with lanes 2 and 3). As a control, the mutant eRF3 KAKA protein associates with eRF1 with similar affinity as wild-type eRF3, suggesting that these mutations do not cause gross structural alterations, although local changes cannot be ruled out. Thus, hUpf1 and PABPC1 interact with a similar, though not necessarily overlapping region of eRF3. The ability of PABPC1 to antagonize the association between hUpf1 and eRF3 in vitro could therefore be a result of a direct competition for eRF3 binding, or of a local structural alteration of eRF3 upon PABPC1 binding, which prevents hUpf1 association.

## Discussion

**Figure 7:**
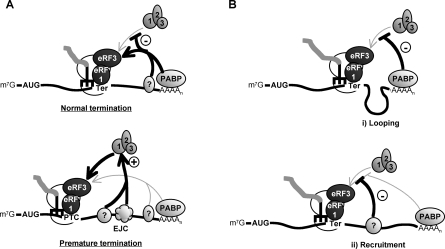
A Competition between Stimulators and Antagonists of Upf Complex Recruitment in Human NMD (A) A unified model where NMD is determined by the balance between 3′ UTR–associated factors that stimulate (such as the EJC) or antagonize (such as cytoplasmic PABP) recruitment of the hUpf complex (shown as spheres labeled 1–3) to the terminating ribosome. (B) Mechanisms by which mammalian mRNAs with long 3′ UTRs may evade NMD (see Discussion for details).

Previous contrasting models for PTC-recognition in NMD invoke either 3′ UTR–associated factors that stimulate NMD, the EJC in human cells [2,44], and DSE-binding proteins in yeast [21], or factors that stimulate normal translation termination and antagonize NMD [1,45]. Our observations, together with the observations in the paper by Eberle et al. [46], are consistent with a unified model for human NMD, in which the balance between NMD-antagonizing (such as PABPC1) and NMD-stimulating (such as the EJC) factor(s) that are associated with the mRNA 3′ UTR, determines whether termination is considered normal or premature (Figure 7A). According to this model, a translation termination event proximal to cytoplasmic PABP (Figure 3), or other unknown NMD-antagonizing factors, precludes the interaction of hUpf1 with eRF3 (Figure 6C) and thus prohibits NMD (Figure 7A, top). By contrast, if hUpf1 associates with eRF3, NMD ensues (Figure 7A, bottom). This occurs when cytoplasmic PABP, or other inhibitory factors, are spatially distant from the termination event (Figure 2) and is enhanced when a splicing event downstream of a termination codon results in deposition of an EJC (Figure 5), which provides higher affinity for the hUpf complex (Figure 7A, bottom). However, an exon-exon junction in the 3′ UTR is not sufficient for NMD (Figure 1). This suggests that a proximal cytoplasmic PABP is dominant over 3′ UTR exon-exon junctions, which is consistent with the observation that the affinity of PABPC1 for eRF3 appears to be several orders of magnitude higher than that of hUpf1 (Figure 6 and unpublished data). However, while introns are observed to only stimulate NMD of the substrates tested in this study, it cannot be ruled out that a subset of human mRNAs requires downstream introns for NMD. Previous experiments, in which EJC or hUpf proteins tethered to an mRNA 3′ UTR were observed to trigger NMD, may have been assisted by the extended 3′ UTRs resulting from insertion of multiple tethering sites and/or by the recruitment of multiple NMD-promoting factors [15,17,47–49]. The model depicted in Figure 7A may be extended to eukaryotes other than mammals and is consistent with the observation in *Drosophila* S2 cells that the decay of an NMD reporter mRNA is inhibited upon cytoplasmic PABP depletion [5]. In this case it is predicted that a large subset of normally stable endogenous mRNAs become NMD substrates, thus out-titrating the NMD pathway.

How does cytoplasmic PABP antagonize NMD? While PABPC1 can out-compete the association of hUpf1 with eRF3 in vitro (Figure 6B), a more complex relationship may exist between these proteins in the cell. For example, we failed to observe exogenously expressed PABPC1 out-compete the co-IP of endogenous hUpf1 with eRF3 (unpublished data). Moreover, in S. cerevisiae, cytoplasmic PABP truncated of its C-terminal eRF3-interaction region was capable of suppressing NMD when tethered in proximity of a PTC [7]. However, we found no loss of eRF3-association of a similarly truncated PABPC1 in co-IP assays between exogenously expressed human proteins (unpublished data), suggesting that eRF3 may form a complex with PABPC1 through additional regions. Understanding the specific mechanism by which NMD is antagonized by cytoplasmic PABP, and likely other 3′ UTR–associated factors, is an important goal for future studies and could involve both direct competition with the Upf complex as well as modulation of the translation termination event that excludes Upf complex recruitment in a more indirect manner. Another open question is how the interplay between eRF3, PABP, and the Upf complex influences events downstream of translation termination. Interestingly, it was previously observed that the interaction between eRF3 and cytoplasmic PABP stimulates mRNA deadenylation in yeast [50], and that deadenylation can be an early step in NMD [51–53]. Clearly, a great deal remains to be learned about the relationship between eRF3, the Upf complex, and cytoplasmic PABP and how it controls the fates of mRNAs after translation termination.

It is likely that 3′ UTR–associated factors (indicated by a question mark in Figure 7A) other than cytoplasmic PABP can antagonize NMD. This hypothesis is consistent with the observation that in yeast cells, cytoplasmic PABP is not required for discriminating tested NMD substrates from their normal counterparts [11]. An excellent candidate for such an activity is the yeast protein Pub1p, which has been identified as a factor that binds downstream of upstream open reading frames (uORFs) in GCN4 and YAP1 mRNAs to prevent NMD [54]. It is possible that Pub1p and factors with similar activities are found in a subset of normal 3′ UTRs. It remains to be tested whether Pub1p acts on the terminating ribosome in a manner similar to cytoplasmic PABP. Similarly, factors other than the EJC could provide an enhanced affinity for the Upf complex and stimulate NMD. For example, the protein Hrp1p appears to serve such a role in the yeast PGK1 NMD substrate [21]. Moreover, human Staufen1 and histone mRNA stem loop binding protein have been shown to recruit hUpf1 to the 3′ UTR of specific mRNAs to trigger NMD-like mRNA decay [55,56]. Thus, our observations suggest that the NMD pathway is much more conserved between mammals and other eukaryotes than previously appreciated. Nevertheless, there is evidence that differences exist between yeast and mammalian cells as to which round of translation can stimulate NMD [28,57–59].

Our observations suggest that while artificial long 3′ UTRs trigger NMD (Figure 2), a subset of mRNAs containing long 3′ UTRs have evolved mechanisms to evade NMD (Figure 4). Future studies should reveal the mechanism by which this is accomplished. This could conceivably be achieved by (i) induced looping of the 3′ UTR, thus placing the poly(A) tail and cytoplasmic PABP in close spatial proximity to the translation termination event (Figure 7B, top), or (ii) by recruitment of factors that antagonize NMD (such as PABPC1 or Pub1p) to the 3′ UTR in proximity to the termination codon (Figure 7B, bottom). The observation that cytoplasmic PABP alleviates NMD when placed in the vicinity of a PTC (Figure 3) [5,7,46] and the finding in the paper by Eberle et al. that artificially induced 3′ UTR looping rescues reporter mRNAs with extended 3′ UTRs from NMD [46], provides proof-of-principle evidence for each of these models. The mechanism by which specific mRNAs evade the NMD pathway is an important subject for future investigation and is likely to vary between individual mRNAs.

After the submission of this paper, we have become aware of two other studies reporting that cytoplasmic PABP antagonizes human NMD when placed in proximity to a PTC [60,61].

## Materials and Methods

### Plasmid constructs.

All plasmid sequences are available upon request. Plasmids expressing different β-globin reporter mRNAs were derived from the pcTET2-βwt plasmid that was constructed by inserting the human β-globin gene between HindIII and ApaI sites of a pcDNA3-based plasmid containing six copies of the Tet-operator sequences upstream of the TATA box. For extended 3′ UTR constructs, parts of the GAPDH mRNA coding sequence and the entire GAPDH 3′ UTR (pcTET2-βGAP) or the GFP ORF (pcTET2-βGFP) were inserted between NotI and XbaI sites of the pcTET2-βwt plasmid, thus replacing the β-globin 3′ UTR. The β-globin stop codon was mutated to UAC by site-directed mutagenesis to generate pcTET2-βGAP-UAC and pcTET2-βGFP-UAC. Plasmids expressing βGAP-UAC-696, βGAP-UAC-485, and βGAP-UAC-422 mRNAs were generated by site-directed mutagenesis of pcTET2-βGAP-UAC to introduce a stop codon (UAA) in the GAPDH sequence, respectively, 696, 485, or 422 nucleotides upstream of the polyadenylation site. The plasmid expressing βwt mRNA was described earlier [47]. To construct plasmids expressing β39–2xMS2-Ex2, β39–2xMS2-Ex3, and β39–2xMS2-3UTR, the 2xMS2 cassette from the previously described plasmid pcβ-2bs [47] was inserted into the BamHI, EcoRI, or NotI sites, respectively, of the pPC-β39 plasmid described earlier [62]. A stretch of A_30_ (pPC-β39-A_30_) or N_30_ (pPC-β39-N_30_) was inserted into the BamHI site of pPC-β39 plasmid using annealed DNA oligos. Similarly, A_30_ (pcTET2-βGAP-A_30_) or N_30_ (pcTET2-βGAP-N_30_) was inserted into the XbaI site of the pcTET2-βGAP plasmid. To construct the pcTET2-βGAP-4xMS2 plasmid, four MS2 binding sites were amplified from a previously described plasmid pcβ-4bs [47] and inserted into the XbaI site of pcTET2-βGAP. Plasmids expressing βAdML, βAdML-UAC, and βTPIi6 mRNAs were constructed by cloning the AdML intron or TPI intron 6 (TPIi6) and flanking exon sequences into the XbaI site in pcTET2-βwt or pcTET2-βwt-UAC plasmids. βGAP-AdML and βAdML-GAP mRNA– expressing plasmids were constructed by inserting the same AdML intron into XbaI and EcoRI sites, respectively, in the pcTET2-βwtGAP plasmid. Plasmids expressing chimeric β-globin mRNAs with 3′ UTRs from Smg5, Cript1, and Tram1 genes, the respective 3′ UTRs, were cloned into the NotI-XbaI sites of pcTET2-βwt.

Plasmids expressing TPI reporter mRNAs were constructed by inserting the entire human TPI gene between HindIII and XbaI sites of the pcTET2 plasmid. A NotI site was inserted into exon 6 (in a manner that preserved the encoded protein) by site-directed mutagenesis. Codon 189 was mutated to TGA using site-directed mutagenesis to generate pcTET2-TPI-189. To remove intron 6, a NotI-XbaI fragment containing exon6-intron6-exon7 was replaced by the same region amplified from TPI cDNA, to generate pcTET2-TPIΔi6–189. To extend the TPI 3′ UTR, a fragment containing part of the GAPDH coding region and 3′ UTR was inserted into the NotI site of pcTET2-TPIΔi6–189 to generate pcTET2-TPIΔi6–189-GAP, or into the NotI site of pcTET2-TPIΔi6 to give rise to pcTET2-TPIΔi6-GAP. TPI-AdML mRNA–expressing plasmid was constructed by inserting the AdML intron and flanking exonic sequences into the XbaI site of pcTET2-TPI.

The plasmid expressing intron-containing GPx1 mRNA with a PTC (pPC-GPx1-UAA) was described earlier [18]. GPx1 cDNA (HindIII-XbaI) sequence replaced the intron-containing sequence in pPC-GPx1Δi-UAA.

The constructs for knockdowns were based on the pSHAG plasmid (a gift from Dr. G. Hannon) and contained inserts expressing precursors to hUpf1, hUpf2, or eIF4AIII siRNAs described earlier [63,64].

Plasmids expressing FLAG-hUpf1, FLAG-PABPC1, FLAG-hnRNP A1, and Myc-hnRNP A1 were described earlier [47,62]. pcDNA3-Myc-eRF3 was constructed by inserting the ORF of eRF3 (longer isoform) between BamHI and NotI sites of the pcDNA3-Myc vector previously described [65]. pcDNA3-Myc-eRF3 KAKA was prepared using site-directed mutagenesis (the mutations are: L66K, N69A, A70K, F73A). pcDNA3-MS2-FLAG-PABPC1 or pcDNA3-MS2-FLAG-PABPN1 were obtained by inserting PABPC1 and PABPN1 cDNAs, respectively, into BamHI-NotI sites of pcDNA3-MS2-FLAG described previously [62].

### NMD factor knockdown.

NMD factor knockdowns were performed by co-transfecting cells with reporter mRNA plasmids and plasmids encoding small hairpin (sh)RNAs targeting hUpf1, hUp2, or eIF4AIII, 60 h before pulse-chase mRNA decay assays were carried out.

### mRNA decay assays and Northern blots.

mRNA decay assays were performed in HeLa Tet-off cells in DMEM/10% FBS/tetracycline (50 ng/ml) transfected with β-globin mRNA expression plasmids. For each 2-cm well of HeLa Tet-off cells, 10 ng of pcβG or pcβwt (as an internal control) and 0.2 μg of tetracycline-regulated reporter mRNA expression plasmids were co-transfected using TransIT HeLa Monster reagent (Mirus). For knockdowns, 0.5 μg of pSHAG plasmids were co-transfected. In each transfection, empty pcDNA3 vector was added to 1 μg of total plasmid. 36–40 h after transfection, or approximately 60 h in the case of knockdowns, transcription of reporter mRNAs was induced by removal of tetracycline through washing cells with 1 ml of phosphate-buffered saline (PBS) and adding DMEM/10% FBS. 6 h later, transcription was shut off by adding tetracycline to a final concentration of 1 μg/ml. Cells were washed with 1 ml PBS and taken up in 500 μl of TRIzol (Invitrogen) starting 30 min after tetracycline addition (0 min time point), and subsequently at time points indicated in each figure. For analysis of knockdown of endogenous hUpf1, hUpf2, and eIF4AIII, 0.2 μg of the plasmid pSUPERpuro was co-transfected instead of the plasmids expressing β-globin mRNA, and cells were treated and harvested as described earlier [66]. Total cellular RNA was isolated and analyzed by Northern blots as described earlier [47]. The anti-sense RNA probe used for β-globin mRNA detection was described earlier [47]. Northern blots for exogenously expressed TPI mRNAs were probed using UltraHyb reagent following the manufacturer's protocol (Ambion), with a short anti-sense RNA probe complementary to the bovine growth hormone 3′ UTR sequence encoded from the pcDNA3 plasmid. GPx1 mRNAs were probed as described earlier [18].

### Antibodies and Western blots.

Rabbit polyclonal anti-sera raised against eIF4AIII (amino acids 1–41), hUpf1 (amino acids 1–416), hUpf2 (C-terminal 206 amino acids), and hUpf3b (full-length) were described earlier [18,47]. Monoclonal mouse antibodies were commercially obtained (anti-FLAG M2, Sigma; anti-Myc 9B11, Cell Signaling). Monoclonal mouse anti-HuR antibodies were described earlier [67]. Rabbit polyclonal eRF3 (#ab-49878) and mouse monoclonal PABPC1 (#ab-6125–100) antibodies were from Abcam.

### Immunoprecipitation assays.

In immunoprecipitations shown in Figure 6B, HEK 293T cells were transiently transfected in 3.5-cm plates with plasmids expressing FLAG-hUpf1 (0.4 μg), FLAG-PABP1 (0.5 μg), or FLAG-MS2 (0.5 μg), 0.5 μg of plasmid expressing wild-type or mutant Myc-eRF3 and 0.1 μg of pcDNA3-Myc-hnRNP A1. Empty pcDNA3 plasmid was added to each transfection to a total of 2 μg. 36–40 h post-transfection, cells were lysed in 400 μl of hypotonic gentle lysis buffer (10 mM Tris-HCl [pH 7.5], 10 mM NaCl, 2 mM EDTA, 0.5% Triton X-100, 1.0 mM phenylmethylsulfonyl fluoride, 1 μg/ml of aprotinin, and 1 μg/ml of leupeptin) for 10 min on ice. NaCl was added to 150 mM, and RNase A was added to 125 μg/ml. The extracts were incubated on ice for 5 min and cell debris was removed by centrifugation. RNase-treated lysed cell extracts were incubated for 2 h at 4 °C with 40 μl anti-FLAG M2 agarose (Sigma). The beads were washed eight times with NET-2 (50 mM Tris-HCl [pH 7.5], 150 mM NaCl, 0.05% Triton X-100) and the FLAG-tagged protein was eluted off the beads by gently shaking the beads for 2 h at 4 °C in 20 μl of NET-2 containing 200 μg/ml of FLAG peptide. Immunoprecipitates separated by SDS-PAGE were probed with anti-Myc 9B11 monoclonal antibody (Cell Signaling) at a 1:1,000 dilution.

Co-IPs between wild-type or KAKA-mutant eRF3 and eRF1 were performed as described above from the cells co-transfected with 0.5 μg of plasmids expressing FLAG-tagged proteins (eRF3, eRF3-KAKA, or MS2 as control), 0.5 μg of plasmids expressing Myc-eRF1, and 0.1 μg of Myc-hnRNP A1 expressing plasmid. Endogenous eRF3 IPs (Figure 6A) were performed as described above except that ∼2.5 × 10^7^ HeLa cells were lysed in 1 ml hypotonic gentle lysis buffer, and the lysates were incubated with 10 μg of anti-eRF3 rabbit polyclonal antibody (Abcam), or rabbit pre-immune serum as control, pre-conjugated to 5 mg of protein-A sepharose beads (GE Healthcare).

### In vitro competition assay.

Approximately 10^7^ HEK293T cells from a 10-cm plate expressing Myc-eRF3, or Myc-peptide as a negative control, were lysed in 1 ml hypotonic gentle lysis buffer as described above. The RNase A–treated, cleared extracts were subsequently incubated with 40 μl anti-Myc resin (Sigma) at 4 °C for 2–3 h, following which the beads were washed eight times with 1 ml of NET-2 buffer. The beads were divided into eight equal parts, and indicated amounts of FLAG-hUpf1, FLAG-PABP1, or FLAG-hnRNP A1 proteins, which had each been affinity-purified from RNase A–treated HEK293T cell extracts (protein concentrations estimated by comparison in anti-FLAG Western blot to a GST-FLAG fusion protein of known concentration), were incubated in 50 μl of NET-2 supplemented with 0.1 mg/ml BSA and 0.2 mg/ml FLAG peptide. The reactions were gently shaken at 4 °C for 2–3 h following which the beads were washed eight times with 1 ml of NET-2 buffer. The beads were resuspended in 25 μl of SDS-loading buffer (10 mM Tris-HCl [pH 6.8], 2% SDS, 10% glycerol, 0.5% bromophenol blue, and 50 mM DTT), and 10 μl of the protein sample was resolved on SDS-PAGE followed by Western blot analysis using anti-FLAG M2 antibody (Sigma, 1:1,000 dilution).

## Supporting Information

Figure S1 Knockdown Efficiencies of NMD and EJC Factors Western blots showing the efficiency of knockdown of hUpf1 (lanes 5 and 11), hUpf2 (lane 6), and eIF4AIII (lane 12). Protein levels are compared to 100%, 50%, 25%, and 10% of cell extract from cells expressing an siRNA against F-Luciferase (lanes 1–4 and 7–10). hUpf3b levels served as a loading control.
(1.4 MB TIF)

Figure S2 An Extended 3′ UTR in TPI mRNA Triggers NMD mRNA decay assays showing decay rates of TPI mRNAs with different length 3′ UTRs, due to insertion of a fragment of GAPDH mRNA (see schematics below), in human HeLa Tet-off cells co-expressing siRNAs targeting hUpf1 or Luciferase (Luc; as a control) as indicated. Constitutively expressed βwt mRNA was used as an internal control for quantification. Numbers indicated above the panels indicate time after transcriptional repression. Schematics on the bottom show the used construct with TPI exons indicated as light-gray bars (not to scale), introns as lines, and GAPDH sequences as dark-gray bars. PTC(189) refers to a PTC at codon 189. Numbers on the right indicate mRNA half-lives (t_1/2_; in minutes) calculated from the shown experiment with the average fold increase and standard deviation over the half-life of TPI-189Δi6-GAP mRNA in the presence of Luc siRNA calculated from three or more experiments given in parentheses below.
(5.1 MB TIF)

Figure S3 βGAP mRNA Is Stabilized by Knockdown of the EJC Factor eIF4AIII mRNA decay assays showing decay rates of the β-globin-derived βGAP mRNA with an extended 3′ UTR in human HeLa Tet-off cells knocked down (using RNAi) for eIF4AIII, or as a negative control, Luciferase (Luc), as indicated on the left of each panel (the knockdown efficiency for eIF4AIII is shown in Figure S1). Constitutively expressed βwt mRNA was used as an internal control for quantification. The mRNA half-lives are shown on the right, and the average fold increase in comparison to the Luc control is given with standard deviation in parentheses below.
(1.6 MB TIF)

Figure S4 Minimal 3′ UTR Length That Can Trigger NMD of β-globin mRNA Northern blots showing the decay rates of βGAP or βGAP-UAC mRNAs with successively shorter 3′ UTRs. The siRNAs co-expressed are indicated on the left. The mRNA half-lives are given on the right in minutes. The schematics of the pre-mRNAs from which the reporter mRNAs are derived are given below each panel with the distance between the termination codon and the poly(A) tail indicated.
(1.1 MB TIF)

Figure S5 Exogenously Expressed PABPs Express at Comparable Levels Western blots showing expression levels of different FLAG-tagged proteins expressed in Figure 3A. Endogenous HuR protein serves as a loading control. The asterisks (*) indicate likely degradation products of MS2-PABPN1.
(1.7 MB TIF)

Figure S6 Proximal Recruitment of PABPC1 Rescues β39 mRNA from NMD More Efficiently as Compared to More Distally Recruited PABPC1 Northern blots showing the decay rates of β39 mRNAs with 2XMS2 binding sites at different positions downstream of the PTC (shown in the schematic below). The exogenously expressed proteins are indicated on the left. The decay rates and fold change as compared to the control (expression of MS2 alone) are given with standard deviation (*n* = 3) on the right.
(5.6 MB TIF)

Figure S7 3′ UTRs of mRNAs Upregulated Upon hUpf1 Knockdown Are Longer than Average Cumulative histograms showing the distribution of the estimated lengths of 83 human intron-less (black solid line) 3′ UTRs from mRNAs upregulated upon hUpf1 knockdown, as compared to the 3′ UTR lengths of 83 randomly selected mRNAs not regulated by hUpf1 (dashed line). The table shows the median 3′ UTR length and percent of mRNAs with 3′ UTRs > 420 nt for upregulated and control mRNAs.
(9.2 MB TIF)

## Abbreviations

AdML intron: adenovirus major late intron
CoIP: co-immunoprecipitation
DSE: downstream sequence element
EJC: exon junction complex
GAPDH: glyceraldehyde 3-phosphate dehydrogenase
GFP: green fluorescent protein
GPx1: glutathione peroxidase 1
NMD: nonsense-mediated mRNA decay
PABP: poly(A)-binding protein
PTC: premature termination codon
RNAi: RNA interference
siRNA: small interfering RNA
TPI: triosephosphate isomerase

## References

[pbio-0060111-b001] Amrani N, Sachs MS, Jacobson A (2006). Early nonsense: mRNA decay solves a translational problem. Nat Rev Mol Cell Biol.

[pbio-0060111-b002] Conti E, Izaurralde E (2005). Nonsense-mediated mRNA decay: molecular insights and mechanistic variations across species. Curr Opin Cell Biol.

[pbio-0060111-b003] Isken O, Maquat LE (2007). Quality control of eukaryotic mRNA: safeguarding cells from abnormal mRNA function. Genes Dev.

[pbio-0060111-b004] Sharifi NA, Dietz HC, Maquat LE (2006). Physiologic substrates and functions for mammalian NMD. Nonsense-mediated mRNA decay.

[pbio-0060111-b005] Behm-Ansmant I, Gatfield D, Rehwinkel J, Hilgers V, Izaurralde E (2007). A conserved role for cytoplasmic poly(A)-binding protein 1 (PABPC1) in nonsense-mediated mRNA decay. EMBO J.

[pbio-0060111-b006] Muhlrad D, Parker R (1999). Aberrant mRNAs with extended 3′ UTRs are substrates for rapid degradation by mRNA surveillance. RNA.

[pbio-0060111-b007] Amrani N, Ganesan R, Kervestin S, Mangus DA, Ghosh S (2004). A faux 3′-UTR promotes aberrant termination and triggers nonsense-mediated mRNA decay. Nature.

[pbio-0060111-b008] Cosson B, Couturier A, Chabelskaya S, Kiktev D, Inge-Vechtomov S (2002). Poly(A)-binding protein acts in translation termination via eukaryotic release factor 3 interaction and does not influence [PSI(+)] propagation. Mol Cell Biol.

[pbio-0060111-b009] Graber JH, McAllister GD, Smith TF (2002). Probabilistic prediction of Saccharomyces cerevisiae mRNA 3′-processing sites. Nucleic Acids Res.

[pbio-0060111-b010] Hoskins RA, Smith CD, Carlson JW, Carvalho AB, Halpern A (2002). Heterochromatic sequences in a Drosophila whole-genome shotgun assembly. Genome Biol.

[pbio-0060111-b011] Meaux S, van Hoof A, Baker KE (2008). Nonsense-mediated mRNA decay in yeast does not require PAB1 or a poly(A) tail. Mol Cell.

[pbio-0060111-b012] Mazumder B, Seshadri V, Fox PL (2003). Translational control by the 3′-UTR: the ends specify the means. Trends Biochem Sci.

[pbio-0060111-b013] Le Hir H, Izaurralde E, Maquat LE, Moore MJ (2000). The spliceosome deposits multiple proteins 20–24 nucleotides upstream of mRNA exon-exon junctions. EMBO J.

[pbio-0060111-b014] Kim VN, Kataoka N, Dreyfuss G (2001). Role of the nonsense-mediated decay factor hUpf3 in the splicing-dependent exon-exon junction complex. Science.

[pbio-0060111-b015] Lykke-Andersen J, Shu MD, Steitz JA (2001). Communication of the position of exon-exon junctions to the mRNA surveillance machinery by the protein RNPS1. Science.

[pbio-0060111-b016] Fribourg S, Gatfield D, Izaurralde E, Conti E (2003). A novel mode of RBD-protein recognition in the Y14-Mago complex. Nat Struct Biol.

[pbio-0060111-b017] Gehring NH, Neu-Yilik G, Schell T, Hentze MW, Kulozik AE (2003). Y14 and hUpf3b form an NMD-activating complex. Mol Cell.

[pbio-0060111-b018] Singh G, Jakob S, Kleedehn MG, Lykke-Andersen J (2007). Communication with the exon-junction complex and activation of nonsense-mediated decay by human Upf proteins occur in the cytoplasm. Mol Cell.

[pbio-0060111-b019] Gatfield D, Unterholzner L, Ciccarelli FD, Bork P, Izaurralde E (2003). Nonsense-mediated mRNA decay in Drosophila: at the intersection of the yeast and mammalian pathways. EMBO J.

[pbio-0060111-b020] Longman D, Plasterk RH, Johnstone IL, Caceres JF (2007). Mechanistic insights and identification of two novel factors in the C. elegans NMD pathway. Genes Dev.

[pbio-0060111-b021] Gonzalez CI, Ruiz-Echevarria MJ, Vasudevan S, Henry MF, Peltz SW (2000). The yeast hnRNP-like protein Hrp1/Nab4 marks a transcript for nonsense-mediated mRNA decay. Mol Cell.

[pbio-0060111-b022] Peltz SW, Brown AH, Jacobson A (1993). mRNA destabilization triggered by premature translational termination depends on at least three *cis*-acting sequence elements and one *trans*-acting factor. Genes Dev.

[pbio-0060111-b023] Thermann R, Neu-Yilik G, Deters A, Frede U, Wehr K (1998). Binary specification of nonsense codons by splicing and cytoplasmic translation. EMBO J.

[pbio-0060111-b024] Nott A, Le Hir H, Moore MJ (2004). Splicing enhances translation in mammalian cells: an additional function of the exon junction complex. Genes Dev.

[pbio-0060111-b025] Wiegand HL, Lu S, Cullen BR (2003). Exon junction complexes mediate the enhancing effect of splicing on mRNA expression. Proc Natl Acad Sci U S A.

[pbio-0060111-b026] Buhler M, Steiner S, Mohn F, Paillusson A, Muhlemann O (2006). EJC-independent degradation of nonsense immunoglobulin-mu mRNA depends on 3′ UTR length. Nat Struct Mol Biol.

[pbio-0060111-b027] Zhang J, Sun X, Qian Y, Maquat LE (1998). Intron function in the nonsense-mediated decay of beta-globin mRNA: indications that pre-mRNA splicing in the nucleus can influence mRNA translation in the cytoplasm. RNA.

[pbio-0060111-b028] Matsuda D, Hosoda N, Kim YK, Maquat LE (2007). Failsafe nonsense-mediated mRNA decay does not detectably target eIF4E-bound mRNA. Nat Struct Mol Biol.

[pbio-0060111-b029] Weil JE, Beemon KL (2006). A 3′ UTR sequence stabilizes termination codons in the unspliced RNA of Rous sarcoma virus. RNA.

[pbio-0060111-b030] Maquat LE, Li X (2001). Mammalian heat shock p70 and histone H4 transcripts, which derive from naturally intronless genes, are immune to nonsense-mediated decay. RNA.

[pbio-0060111-b031] Mangus DA, Evans MC, Jacobson A (2003). Poly(A)-binding proteins: multifunctional scaffolds for the post-transcriptional control of gene expression. Genome Biol.

[pbio-0060111-b032] Mendell JT, Sharifi NA, Meyers JL, Martinez-Murillo F, Dietz HC (2004). Nonsense surveillance regulates expression of diverse classes of mammalian transcripts and mutes genomic noise. Nat Genet.

[pbio-0060111-b033] Pan Q, Saltzman AL, Kim YK, Misquitta C, Shai O (2006). Quantitative microarray profiling provides evidence against widespread coupling of alternative splicing with nonsense-mediated mRNA decay to control gene expression. Genes Dev.

[pbio-0060111-b034] Weng Y, Czaplinski K, Peltz SW (1996). Identification and characterization of mutations in the UPF1 gene that affect nonsense suppression and the formation of the Upf protein complex but not mRNA turnover. Mol Cell Biol.

[pbio-0060111-b035] Czaplinski K, Ruiz-Echevarria MJ, Paushkin SV, Han X, Weng Y (1998). The surveillance complex interacts with the translation release factors to enhance termination and degrade aberrant mRNAs. Genes Dev.

[pbio-0060111-b036] Cosson B, Berkova N, Couturier A, Chabelskaya S, Philippe M (2002). Poly(A)-binding protein and eRF3 are associated in vivo in human and Xenopus cells. Biol Cell.

[pbio-0060111-b037] Kashima I, Yamashita A, Izumi N, Kataoka N, Morishita R (2006). Binding of a novel SMG-1-Upf1-eRF1-eRF3 complex (SURF) to the exon junction complex triggers Upf1 phosphorylation and nonsense-mediated mRNA decay. Genes Dev.

[pbio-0060111-b038] Kozlov G, Trempe JF, Khaleghpour K, Kahvejian A, Ekiel I (2001). Structure and function of the C-terminal PABC domain of human poly(A)-binding protein. Proc Natl Acad Sci U S A.

[pbio-0060111-b039] Uchida N, Hoshino S, Imataka H, Sonenberg N, Katada T (2002). A novel role of the mammalian GSPT/eRF3 associating with poly(A)-binding protein in Cap/Poly(A)-dependent translation. J Biol Chem.

[pbio-0060111-b040] Hoshino S, Hosoda N, Araki Y, Kobayashi T, Uchida N (1999). Novel function of the eukaryotic polypeptide-chain releasing factor 3 (eRF3/GSPT) in the mRNA degradation pathway. Biochemistry (Mosc).

[pbio-0060111-b041] Gorlach M, Burd CG, Dreyfuss G (1994). The mRNA poly(A)-binding protein: localization, abundance, and RNA-binding specificity. Exp Cell Res.

[pbio-0060111-b042] Pal M, Ishigaki Y, Nagy E, Maquat LE (2001). Evidence that phosphorylation of human Upfl protein varies with intracellular location and is mediated by a wortmannin-sensitive and rapamycin-sensitive PI 3-kinase-related kinase signaling pathway. RNA.

[pbio-0060111-b043] Kozlov G, De Crescenzo G, Lim NS, Siddiqui N, Fantus D (2004). Structural basis of ligand recognition by PABC, a highly specific peptide-binding domain found in poly(A)-binding protein and a HECT ubiquitin ligase. EMBO J.

[pbio-0060111-b044] Maquat LE (2005). Nonsense-mediated mRNA decay in mammals. J Cell Sci.

[pbio-0060111-b045] Hilleren P, Parker R (1999). mRNA surveillance in eukaryotes: kinetic proofreading of proper translation termination as assessed by mRNP domain organization. RNA.

[pbio-0060111-b046] Eberle AB, Mathys H, Stalder L, Orozco RZ, Mühlemann O (2008). Posttranscriptional gene regulation by spatial rearrangement of the 3′ untranslated region. PLoS Biol.

[pbio-0060111-b047] Lykke-Andersen J, Shu MD, Steitz JA (2000). Human Upf proteins target an mRNA for nonsense-mediated decay when bound downstream of a termination codon. Cell.

[pbio-0060111-b048] Gehring NH, Kunz JB, Neu-Yilik G, Breit S, Viegas MH (2005). Exon-junction complex components specify distinct routes of nonsense-mediated mRNA decay with differential cofactor requirements. Mol Cell.

[pbio-0060111-b049] Unterholzner L, Izaurralde E (2004). SMG7 acts as a molecular link between mRNA surveillance and mRNA decay. Mol Cell.

[pbio-0060111-b050] Hosoda N, Kobayashi T, Uchida N, Funakoshi Y, Kikuchi Y (2003). Translation termination factor eRF3 mediates mRNA decay through the regulation of deadenylation. J Biol Chem.

[pbio-0060111-b051] Cao D, Parker R (2003). Computational modeling and experimental analysis of nonsense-mediated decay in yeast. Cell.

[pbio-0060111-b052] Chen CY, Shyu AB (2003). Rapid deadenylation triggered by a nonsense codon precedes decay of the RNA body in a mammalian cytoplasmic nonsense-mediated decay pathway. Mol Cell Biol.

[pbio-0060111-b053] Mitchell P, Tollervey D (2003). An NMD pathway in yeast involving accelerated deadenylation and exosome-mediated 3′–>5′ degradation. Mol Cell.

[pbio-0060111-b054] Ruiz-Echevarria MJ, Peltz SW (2000). The RNA binding protein Pub1 modulates the stability of transcripts containing upstream open reading frames. Cell.

[pbio-0060111-b055] Kim YK, Furic L, Desgroseillers L, Maquat LE (2005). Mammalian Staufen1 recruits Upf1 to specific mRNA 3′ UTRs so as to elicit mRNA decay. Cell.

[pbio-0060111-b056] Kaygun H, Marzluff WF (2005). Regulated degradation of replication-dependent histone mRNAs requires both ATR and Upf1. Nat Struct Mol Biol.

[pbio-0060111-b057] Maderazo AB, Belk JP, He F, Jacobson A (2003). Nonsense-containing mRNAs that accumulate in the absence of a functional nonsense-mediated mRNA decay pathway are destabilized rapidly upon its restitution. Mol Cell Biol.

[pbio-0060111-b058] Ishigaki Y, Li X, Serin G, Maquat LE (2001). Evidence for a pioneer round of mRNA translation: mRNAs subject to nonsense-mediated decay in mammalian cells are bound by CBP80 and CBP20. Cell.

[pbio-0060111-b059] Gao Q, Das B, Sherman F, Maquat LE (2005). Cap-binding protein 1-mediated and eukaryotic translation initiation factor 4E-mediated pioneer rounds of translation in yeast. Proc Natl Acad Sci U S A.

[pbio-0060111-b060] Ivanov PV, Gehring NH, Kunz JB, Hentze MW, Kulozik AE (2008). Interactions between UPF1, eRFs, PABP, and the exon junction complex suggest an integrated model for mammalian NMD pathways. EMBO J.

[pbio-0060111-b061] Silva AL, Ribeiro P, Inacio A, Liebhaber SA, Romao L (2008). Proximity of the poly(A)-binding protein to a premature termination codon inhibits mammalian nonsense-mediated mRNA decay. RNA.

[pbio-0060111-b062] Lykke-Andersen J, Wagner E (2005). Recruitment and activation of mRNA decay enzymes by two ARE-mediated decay activation domains in the proteins TTP and BRF-1. Genes Dev.

[pbio-0060111-b063] Mendell JT, ap Rhys CM, Dietz HC (2002). Separable roles for rent1/hUpf1 in altered splicing and decay of nonsense transcripts. Science.

[pbio-0060111-b064] Palacios IM, Gatfield D, St Johnston D, Izaurralde E (2004). An eIF4AIII-containing complex required for mRNA localization and nonsense-mediated mRNA decay. Nature.

[pbio-0060111-b065] Lykke-Andersen J (2002). Identification of a human decapping complex associated with hUpf proteins in nonsense-mediated decay. Mol Cell Biol.

[pbio-0060111-b066] Paillusson A, Hirschi N, Vallan C, Azzalin CM, Muhlemann O (2005). A GFP-based reporter system to monitor nonsense-mediated mRNA decay. Nucleic Acids Res.

[pbio-0060111-b067] Gallouzi IE, Brennan CM, Stenberg MG, Swanson MS, Eversole A (2000). HuR binding to cytoplasmic mRNA is perturbed by heat shock. Proc Natl Acad Sci U S A.

