# Supplementary figures and images for: A Competition between Stimulators and Antagonists of Upf Complex Recruitment Governs Human Nonsense-Mediated mRNA Decay
Source: PLoS Biol. 2008 Apr 29;6(4):e111. doi: 10.1371/journal.pbio.0060111 (PMC2689706; doi:10.1371/journal.pbio.0060111)

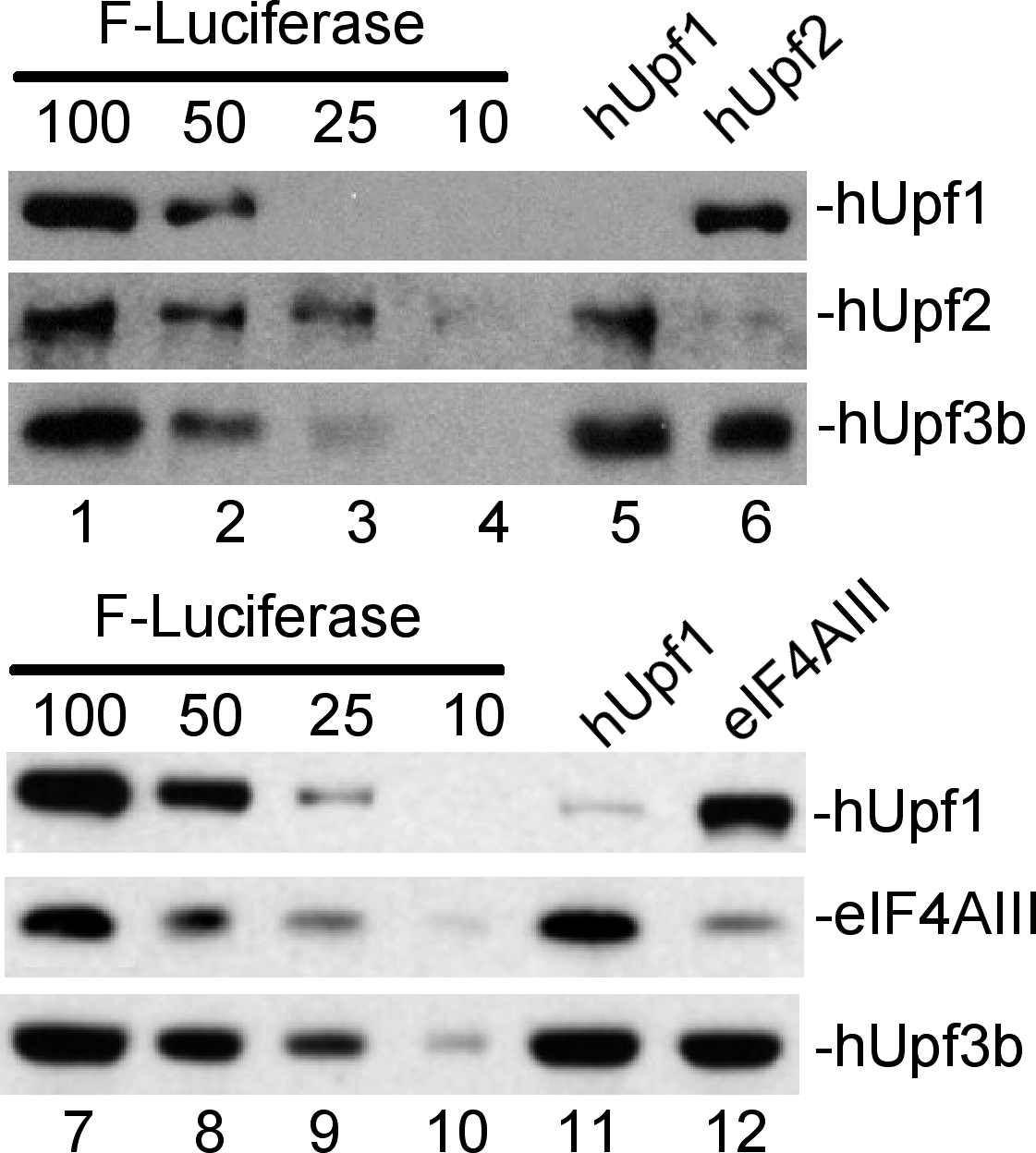

Supplement: Figure S1 — Western blots showing the efficiency of knockdown of hUpf1 (lanes 5 and 11), hUpf2 (lane 6), and eIF4AIII (lane 12). Protein levels are compared to 100%, 50%, 25%, and 10% of cell extract from cells expressing an siRNA against F-Luciferase (lanes 1–4 and 7–10). hUpf3b levels served as a loading control. (1.4 MB TIF) [file pbio.0060111.sg001.tif]

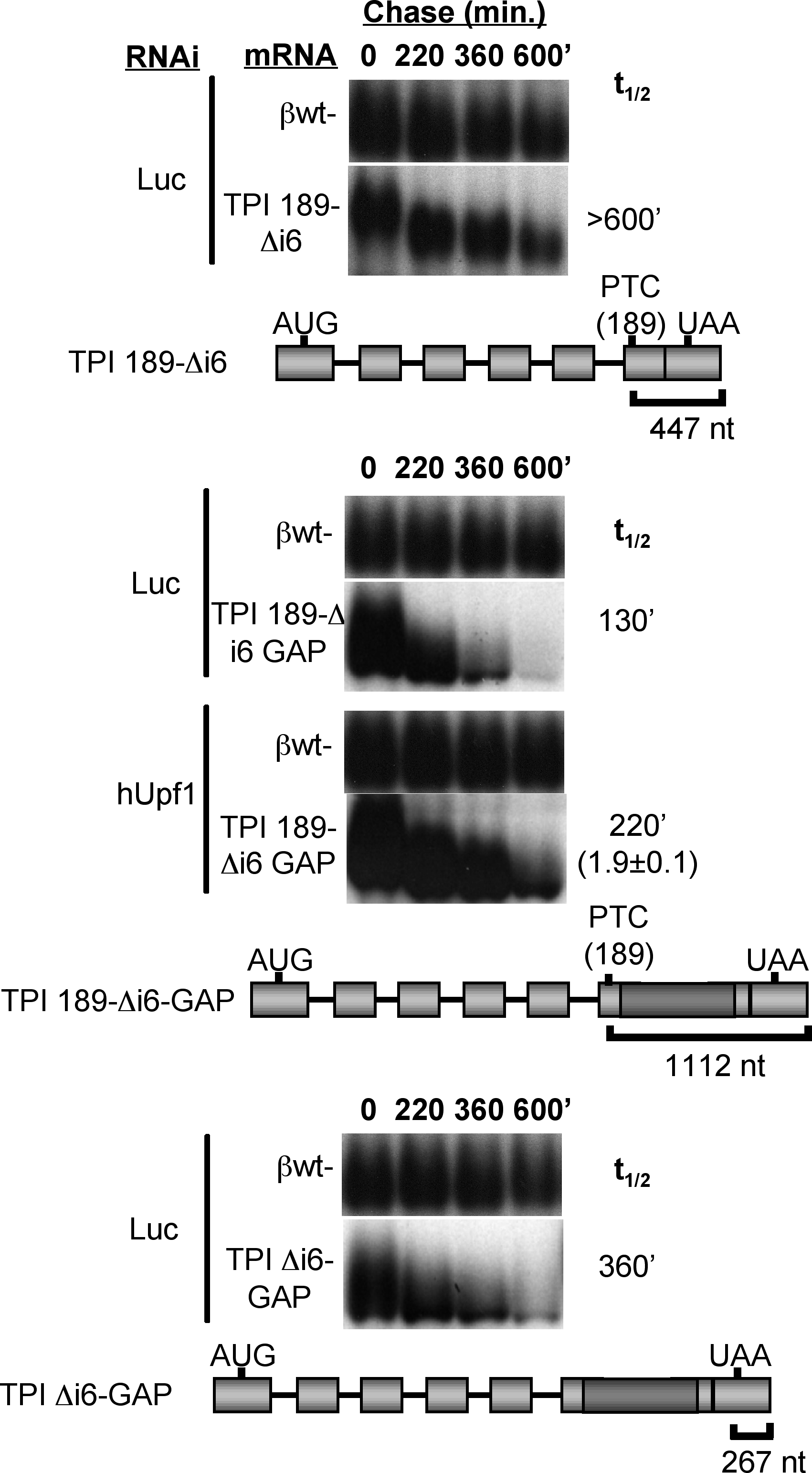

Supplement: Figure S2 — mRNA decay assays showing decay rates of TPI mRNAs with different length 3′ UTRs, due to insertion of a fragment of GAPDH mRNA (see schematics below), in human HeLa Tet-off cells co-expressing siRNAs targeting hUpf1 or Luciferase (Luc; as a control) as indicated. Constitutively expressed βwt mRNA was used as an internal control for quantification. Numbers indicated above the panels indicate time after transcriptional repression. Schematics on the bottom show the used construct with TPI exons indicated as light-gray bars (not to scale), introns as lines, and GAPDH sequences as dark-gray bars. PTC(189) refers to a PTC at codon 189. Numbers on the right indicate mRNA half-lives (t1/2; in minutes) calculated from the shown experiment with the average fold increase and standard deviation over the half-life of TPI-189Δi6-GAP mRNA in the presence of Luc siRNA calculated from three or more experiments given in parentheses below. (5.1 MB TIF) [file pbio.0060111.sg002.tif]

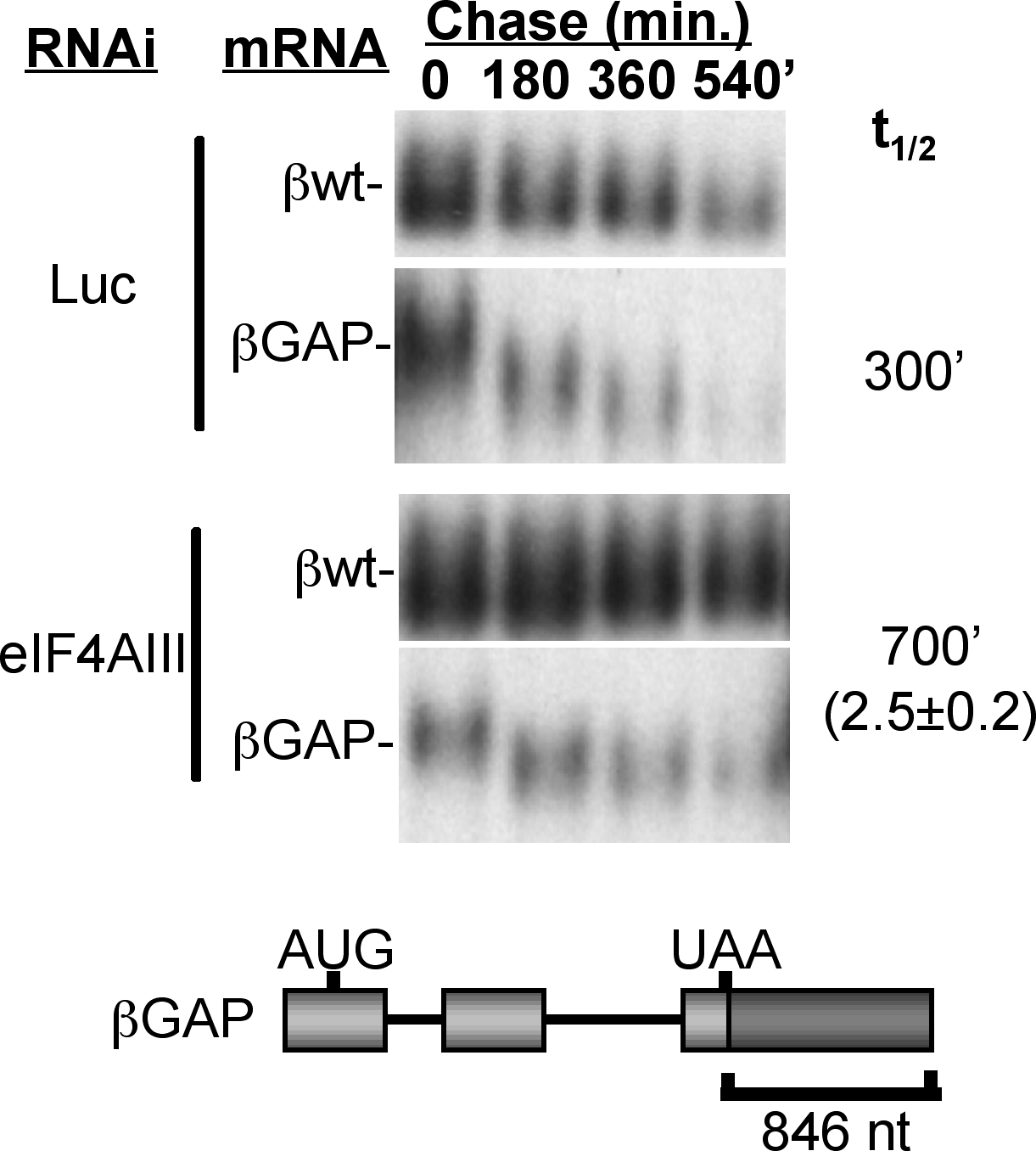

Supplement: Figure S3 — mRNA decay assays showing decay rates of the β-globin-derived βGAP mRNA with an extended 3′ UTR in human HeLa Tet-off cells knocked down (using RNAi) for eIF4AIII, or as a negative control, Luciferase (Luc), as indicated on the left of each panel (the knockdown efficiency for eIF4AIII is shown in Figure S1). Constitutively expressed βwt mRNA was used as an internal control for quantification. The mRNA half-lives are shown on the right, and the average fold increase in comparison to the Luc control is given with standard deviation in parentheses below. (1.6 MB TIF) [file pbio.0060111.sg003.tif]

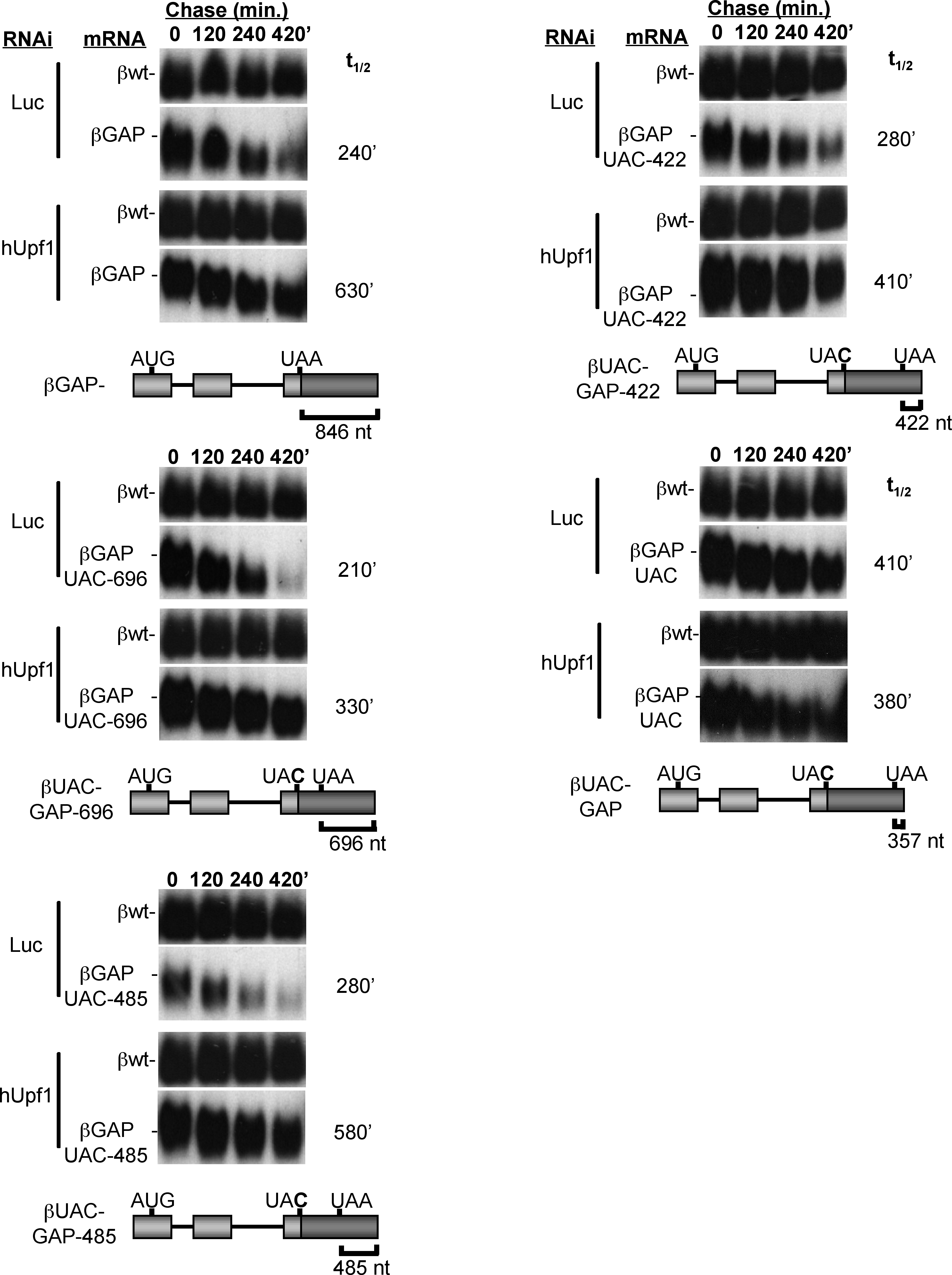

Supplement: Figure S4 — Northern blots showing the decay rates of βGAP or βGAP-UAC mRNAs with successively shorter 3′ UTRs. The siRNAs co-expressed are indicated on the left. The mRNA half-lives are given on the right in minutes. The schematics of the pre-mRNAs from which the reporter mRNAs are derived are given below each panel with the distance between the termination codon and the poly(A) tail indicated. (1.1 MB TIF) [file pbio.0060111.sg004.tif]

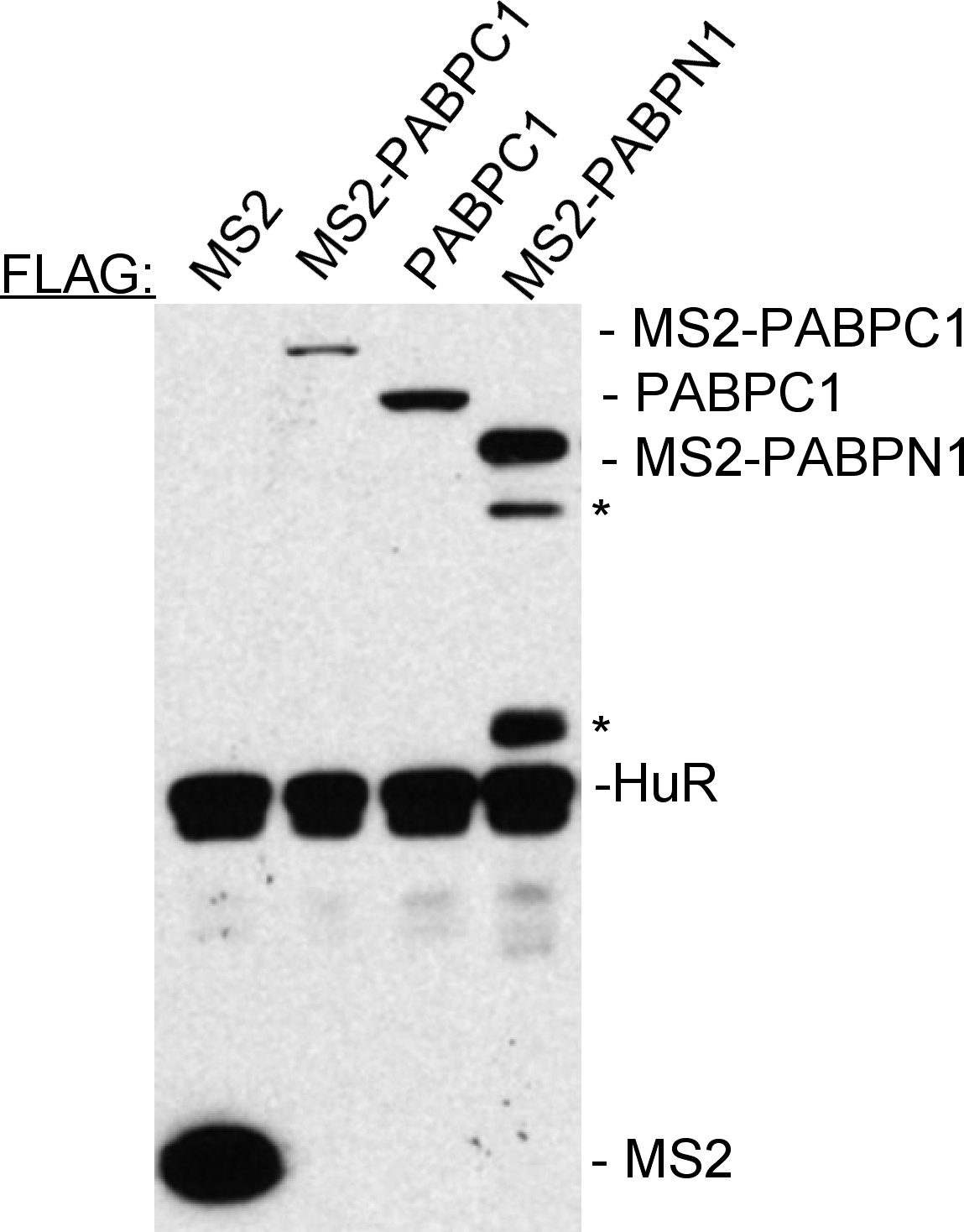

Supplement: Figure S5 — Western blots showing expression levels of different FLAG-tagged proteins expressed in Figure 3A. Endogenous HuR protein serves as a loading control. The asterisks (*) indicate likely degradation products of MS2-PABPN1. (1.7 MB TIF) [file pbio.0060111.sg005.tif]

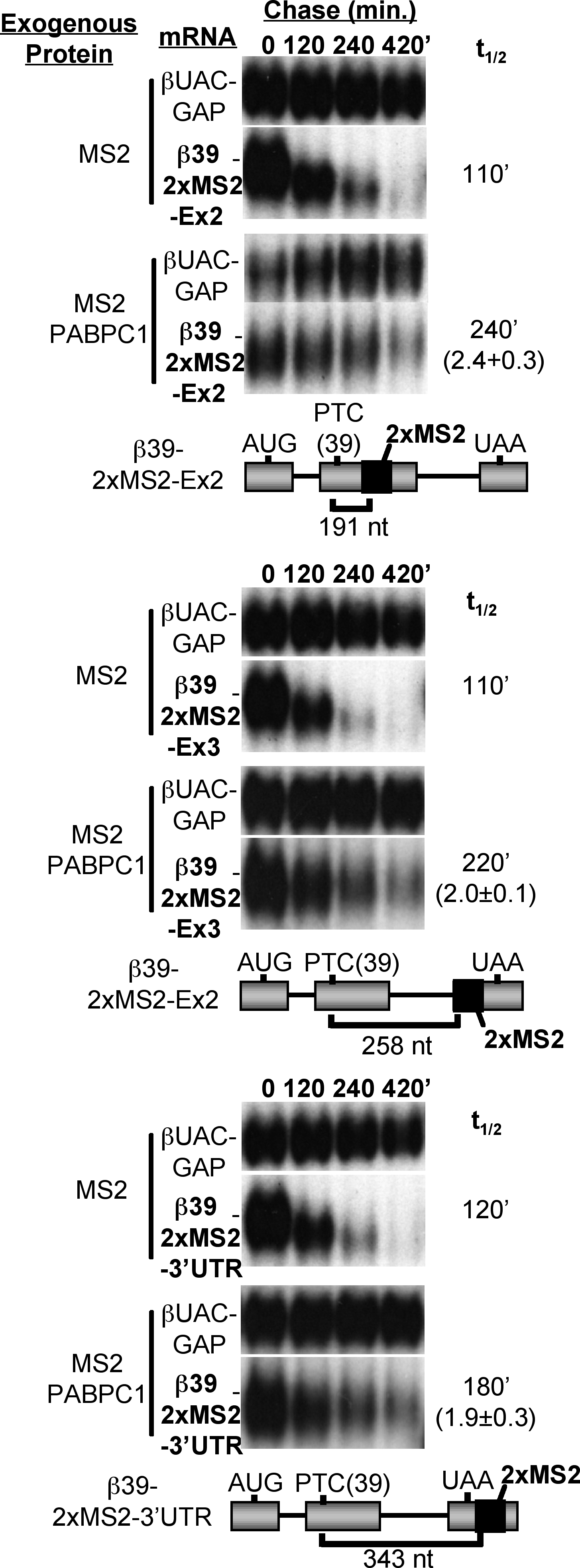

Supplement: Figure S6 — Northern blots showing the decay rates of β39 mRNAs with 2XMS2 binding sites at different positions downstream of the PTC (shown in the schematic below). The exogenously expressed proteins are indicated on the left. The decay rates and fold change as compared to the control (expression of MS2 alone) are given with standard deviation (n = 3) on the right. (5.6 MB TIF) [file pbio.0060111.sg006.tif]

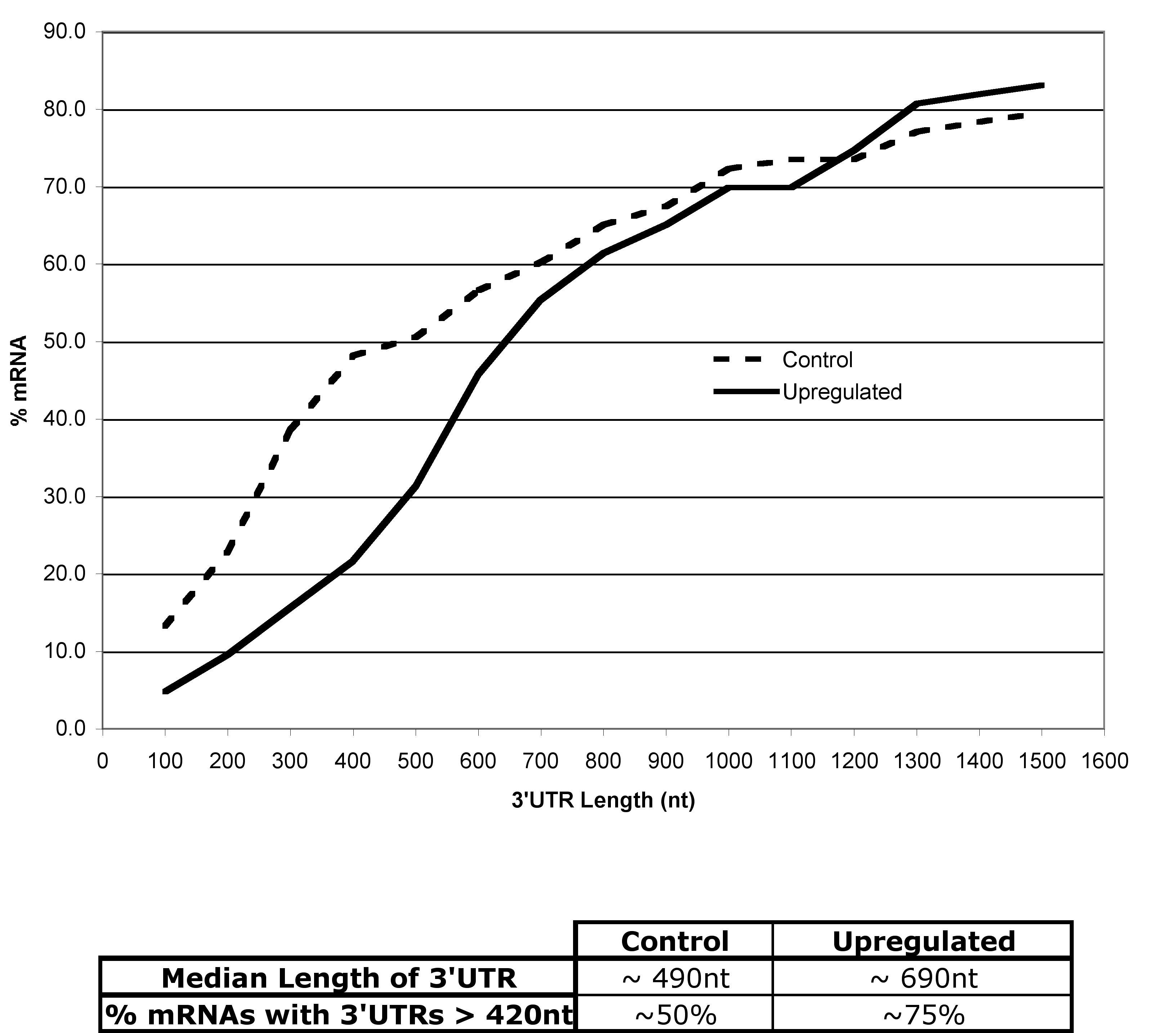

Supplement: Figure S7 — Cumulative histograms showing the distribution of the estimated lengths of 83 human intron-less (black solid line) 3′ UTRs from mRNAs upregulated upon hUpf1 knockdown, as compared to the 3′ UTR lengths of 83 randomly selected mRNAs not regulated by hUpf1 (dashed line). The table shows the median 3′ UTR length and percent of mRNAs with 3′ UTRs > 420 nt for upregulated and control mRNAs. (9.2 MB TIF) [file pbio.0060111.sg007.tif]
